# SRCP: a comprehensive pipeline for accurate annotation and quantification of circRNAs

**DOI:** 10.1186/s13059-021-02497-7

**Published:** 2021-09-23

**Authors:** Avigayel Rabin, Michela Zaffagni, Reut Ashwal-Fluss, Ines Lucia Patop, Aarti Jajoo, Shlomo Shenzis, Liran Carmel, Sebastian Kadener

**Affiliations:** 1 Biological Chemistry Department, Silberman Institute of Life Sciences, The Hebrew University of Jerusalem, 91904 Jerusalem, Israel; 2 Biology Department, Brandeis University, Waltham, MA 02454 USA; 3 Department of Genetics, Silberman Institute of Life Sciences, The Hebrew University of Jerusalem, 91904 Jerusalem, Israel

**Keywords:** Circular RNA, CircRNAs, RNA metabolism, Splicing, Pipeline, AGO2

## Abstract

Here we describe a new integrative approach for accurate annotation and quantification of circRNAs named Short Read circRNA Pipeline (SRCP). Our strategy involves two steps: annotation of validated circRNAs followed by a quantification step. We show that SRCP is more sensitive than other individual pipelines and allows for more comprehensive quantification of a larger number of differentially expressed circRNAs. To facilitate the use of SRCP, we generate a comprehensive collection of validated circRNAs in five different organisms, including humans. We then utilize our approach and identify a subset of circRNAs bound to the miRNA-effector protein AGO2 in human brain samples.

**Supplementary Information:**

The online version contains supplementary material available at 10.1186/s13059-021-02497-7.

## Background

Circular RNAs (circRNAs) are abundant RNAs generated through circularization of specific exons by a process called back splicing [1–4]. circRNAs have been found in bacteria, archaea, and most eukaryotes [5]. While circRNAs are produced by splicing in most eukaryotes, it is not clear how are they produced in bacteria and archaea [4]. Because circRNAs are covalently closed molecules, they are generally more stable than linear RNA transcripts, as they cannot be efficiently target by the canonical mRNA degradation pathways (e.g., exonucleases digestion). circRNAs are highly expressed in metazoans, particularly in the central nervous system (CNS) [1, 6–8]. Interestingly, circRNAs accumulate in the CNS as animals age in flies, worms, and mice [9, 10]. When first discovered, circRNAs were thought to be a byproduct of splicing; however, multiple studies in the past years have clearly shown that at least some of these RNAs are functional. Two pioneering works showed that circRNAs can bind to and likely modulate miRNA function [11, 12]. Other studies showed that these molecules can also regulate the activity of RNA-binding proteins [13] and ribosome biogenesis [14] and that a subset of them encode proteins [15–17]. Their functionality has also been demonstrated in vivo [15, 18–20], some of them are altered in disease [21] and there is evidence that circRNAs can be used as disease biomarkers [22].

Many computational pipelines exist for de novo discovery and quantification of circRNAs from RNA-seq data [23–25]. These include Acfs [26], DCC [27], segemehl [28], CIRCexplorer [29], KNIFE [30], MapSplice2 [31], circRNA_finder [32], CIRI [33], and find_circ [11]. The pipelines differ in sensitivity, precision, runtime, and storage requirements [23], as shown in several independent studies [23, 34, 35]. Analysis of a large number of datasets allowed researchers to generate different circRNA databases [36]. One of the most popular circRNAs databases is known as circBASE [37], which contains annotations and information on many, but not all, circRNAs. Since many circRNAs have been already discovered, the very time-consuming [23] process of de novo identification of circRNAs for every RNA-seq library is redundant. Moreover, most circRNAs are identified by only a subset or even only one pipeline, making it difficult to determine whether these are real circRNAs or sequencing and/or annotation artifacts. Therefore, utilizing only one pipeline for circRNA annotation and quantification is highly problematic [35].

Generally, circRNA detection relies on the identification of a splicing junction that is unique for a given circRNA (i.e., the backsplicing junction). Importantly, other biological processes and technical artifacts can generate splicing junctions that can be confused with those characteristic of circRNAs [25]. These include splicing errors, trans-splicing, linear concatamers (i.e., from exon duplications), and artifacts resulting from the template switching activity of the reverse transcriptase [25]. Hence, circRNAs need to be validated experimentally. To do so, researchers use the 3′ exonuclease RNaseR, which efficiently degrades linear RNA sequences and does not affect circular RNA molecules [38]. Briefly, the usual approach consists in comparing the abundance of specific splicing junctions (determined by RT-PCR or RNA-seq) in samples pretreated or not with RNaseR. Although this is the most straightforward approach for validation of candidate backsplicing junctions, it cannot be used as sole evidence of RNA circularity, as some linear RNAs are resistant to RNaseR treatment [39, 40]. In sum, current pipelines are useful for annotating and quantifying specific sets of circRNAs. However, utilizing only one pipeline results in large numbers of false positives and false negatives. While combining several pipelines could help to identify all bona fide circRNAs, the quantification approaches are different, and their results cannot be merged. Moreover, to perform quantification of circRNAs, most pipelines rely on the de novo annotation of circRNAs. The redundant de novo annotation is a very time consuming and computationally intensive process.

Here, we developed an alternative approach for both identifying and quantifying circRNAs. We first generate a reliable database of circRNAs by analyzing RNA-seq data from RNaseR and mock-treated samples through several existing pipelines. In the second step, we use this database to create a reference to which the RNA-seq data is aligned. We showed that our approach outperforms the use of single pipelines with regard to both annotation and quantification. Then, we generated, sequenced, and analyzed mock and RNaseR-treated samples from mouse, rat, monkey, and human tissues. This allowed us to create a catalog of bona fide circRNAs for utilizing our new pipeline in those species. Finally, we used this catalog to identify circRNAs bound to the miRNA-effector protein AGO2 in human brain samples.

## Results

### General approach

#### circRNA annotation

**Fig. 1:**
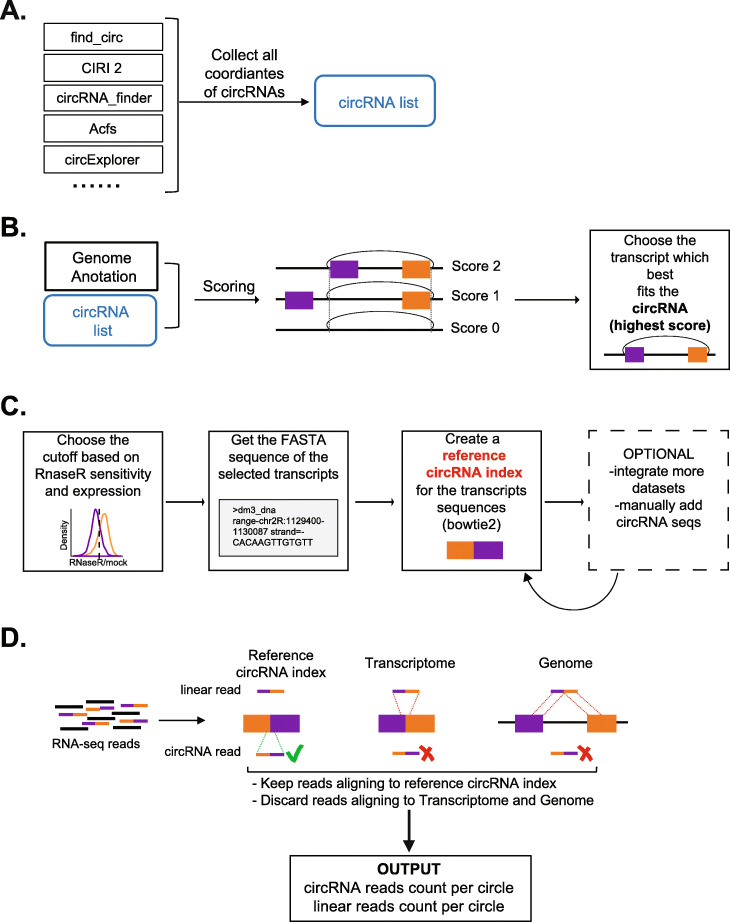
A comprehensive approach for annotation and quantification of circRNAs. **A** As a first step towards using SRCP, we generated a comprehensive list of all possible circRNAs in a given tissue/species. We generated this list by merging circRNA coordinates provided by different pipelines. **B** We then reannotate the initial list to obtain one specific set of coordinates for each candidate circRNA. To do so, we rely on the fact that most circRNAs are flanked by already annotated splice sites. Then, if the start coordinates and the end coordinate of the circRNA are both exactly on a 5′ and 3′ boundaries of the transcript’s exons, we compute a score of 2. (ii) If only one coordinate is exactly on an exon boundary, the score is 1. (iii) If neither coordinate is on any exon boundary, the score is 0. We keep the transcript with the highest score. **C** We determine the cutoff (false-positive and false-negative rate) based on RNaseR sensitivity and expression level. Then we obtain a circRNA index. Importantly, circRNAs from other lists and/or databases can be added to enrich the circRNA index. **D** Once the circRNA index is set up, SRCP allows accurate quantification of circRNA reads

To accurately annotate and quantify circRNAs, we utilize a two-step approach that we call the Short Read CircRNA Pipeline (SRCP; Fig. 1). The first step consists of the annotation of validated circRNAs. To do this, we utilize several pipelines to identify all putative circRNAs in a given tissue/species (Fig. 1A). As annotation of the boundaries of circRNAs can be ambiguous between reads and/or between pipelines, we score the potential boundaries for a circRNA depending on the presence of zero, one, or two annotated spliced junctions (scores of 0, 1, and 2, respectively; Fig. 1B). Then, we proceed to quantify all the putative circRNAs (from the output of all the used pipelines) using SRCP (see below). We perform this quantification in two different RNA-seq libraries: one generated from total rRNA-depleted RNA and one generated from the same RNA pretreated with RNaseR (Fig. 1A, B). The comparison allows us to establish a database containing all validated circRNAs based on RNaseR resistance, as well as on whether they are contained within annotated exons (Fig. 1C). Creating this database is time consuming, but it is only performed once for each type of sample (e.g., tissue and/or organism). Importantly, generating the list of bona fide circRNAs requires the careful determination of a threshold (Fig. 1C). The selection of this cutoff is somehow arbitrary, but the user can change the threshold to include circRNAs that were slightly sensitive to RNaseR but are likely to be real based on other criteria. For example, circRNAs that are detected by several pipelines and are expressed at medium to high levels are usually real, even if slightly sensitive to RNaseR. Here, we utilize these criteria in a combined fashion to choose the cutoff for the RNaseR sensitivity at which a circRNA is consider real (true circRNA) and generate a list of bona fide circRNAs from a given tissue or animal. Our pipeline allows the user to customize the circRNA list to be tested by either changing the threshold or rescuing specific circRNAs from the ones considered false, and even manually provide a subset of potential circRNAs to be quantified by SRCP (Fig. 1C). Furthermore, we combine validated circRNAs from different tissues and replicas to generate a comprehensive list of likely valid circRNAs. The main advantage of SRCP over other pipelines is that it can quantify circRNAs identified by any pipeline. In addition, our pipeline does not require de novo identification and annotation to be generated for each sample/run.

#### circRNA quantification

Following the circRNA annotation step, our pipeline generates a reference of the backsplicing junctions and the canonical (linear) splicing junctions of the first and last exon contained in each circRNAs (Fig. 1D); the latter is used to quantify the linear RNA generated from the circRNA-hosting genes. Once the “annotation” and “index reference” creation steps have been performed, SRCP can quantify the levels of circRNAs and their linear counterparts from a particular set of samples. As stated above, this list can be generated using any cutoff chosen by the user and even include specific circRNAs that the user wants to test. Briefly, first SRCP aligns the RNA-seq reads to the species/tissue reference of the sample to be quantified using bowtie2 to identify circRNA reads in the datasets. Then, we align the backsplicing junction reads to the genome and linear transcriptome and eliminate those that align to one or both of them. This is done to remove reads that cannot be unequivocally assigned to the circRNAs. Following this approach, SRCP allows the quantification of any circRNA junction independent of whether is identified by only one, a few, or all available pipelines for circRNA identification and quantification.

### No current pipeline accurately annotates all circRNAs

**Fig. 2:**
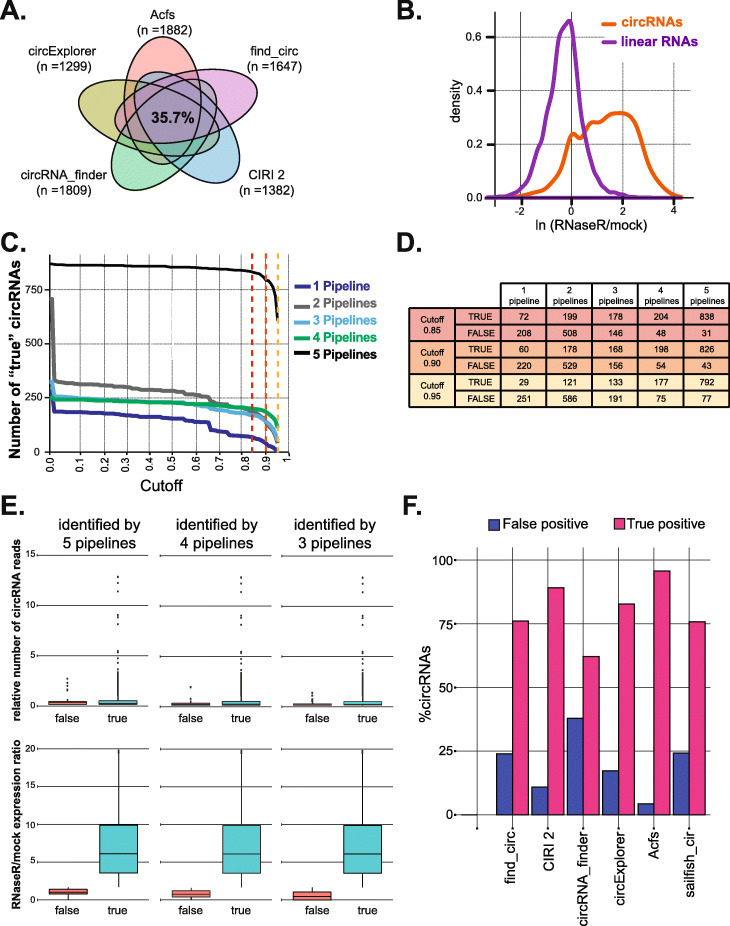
SRCP accurately annotates circRNAs. **A** Venn diagram of the circRNAs found by the circRNA-identification pipelines in analysis of the total RNA library from the GSE55872 dataset. For this and further analysis, we utilized only circRNAs which were found in the mock samples. **B** RNaseR/mock ratio distribution in *Drosophila melanogaster*. The data in orange represent the circular junctions and that in violet the linear junctions. For the linear junctions, we utilized the SRCP output of the mRNAs produced from the genes hosting the potential circRNAs. **C** The number of circRNAs identified as “true” positives as a function of the cutoff for circRNAs identified by 1, 2, 3, 4, or 5 of the pipelines used. The dotted lines indicate three potential threshold/cutoffs (0.85, 0.9, or 0.95 respectively). The cutoff is defined as the fraction of linear mRNAs that would have some resistance to RNaseR. **D** Number of true and false circRNAs that have been identified from 1, 2, 3, 4, or 5 pipelines for different cutoffs in (**C**). **E** Boxplots showing the distribution of expression (top) and the RNaseR/mock ratio (bottom) of the true and false circRNA that are identified either by 3 (right), 4 (middle), or 5 (left) pipelines. **F** Percent of true and false positives identified by SCRP and each individual circRNA-identification pipeline

We began by assessing circRNA levels detected by five commonly utilized circRNA pipelines (find_circ [11], CIRI2 [41], Acfs [26], circExplorer [29], and circRNA_finder [32]) in a previously published *Drosophila melanogaster* RNA-seq dataset obtained on samples with and without RNaseR treatment [GSE55872]. As previously observed for other datasets [23, 35], only about one third of circRNAs were identified by all the tested pipelines (Fig. 2A). It has been previously proposed that a large fraction of pipeline-specific circRNAs are false positives and that circRNAs detected by several pipelines tend to be bona fide circRNAs [5, 35]. More than 50% of the initially identified circRNAs in the analyzed dataset are detected by only one or two pipelines.

### Identification of bona fide circRNAs using RNaseR-seq datasets

The analyzed dataset also contains RNA-seq reads from RNaseR-treated samples, which allowed us to determine the RNaseR sensitivity of the candidate circRNAs, a good way to identify bona fide circRNAs and not computational and/or methodological artifacts. It is important to point out that RNaseR sensitivity is not completely reliable, as linear RNAs with strong secondary structure are resistant to this exonuclease [40]. For each identified circRNA junction, we calculated the ratio between the signal obtained in the RNaseR-treated samples and the signal in the total RNA (RNaseR/mock ratio). For each circRNA candidate, we assumed that the larger the RNaseR/mock ratio, the more likely it represents a bona fide circRNA. For the sake of simplicity and to minimize false positives, we utilized only circRNAs that are expressed in the mock sample.

As expected, linear RNA junctions (from the same locus hosting the circRNAs) have lower RNaseR/mock RNA ratios than circRNA junctions (Fig. 2B). For linear mRNAs, the RNaseR/mock ratios have a discrete peak with a normal distribution. Interestingly, the putative circRNA distribution is broader. This is likely due to overlapping peaks observed in the distribution of the candidate circRNAs. Therefore, it was necessary to apply a cutoff in order to distinguish bona fide circRNAs from false positives. Since false-positive circRNAs are linear molecules, we assumed that the distribution of the linear RNAs and the false-positive circRNAs should be similar. Therefore, we defined the error level to be an arbitrary percentage of the area on the right tail of the linear distribution. We accepted all circRNAs that have a higher RNaseR/mock ratio than this cutoff ratio as real, as this constitutes the barrier for declaring a molecule RNaseR-resistant. The overlapping area of the linear and circular distributions may originate from RNaseR-resistant linear RNA or from real circRNAs with some sensitivity to the RNaseR treatment. The observed population of linear RNAs was quantified using a similar metric (junction reads) but was restricted to the mRNAs generated from the genes hosting the circRNAs. It is important to point out that the cutoff is arbitrary and that SRCP takes as input a list of circRNAs that can be modified either by changing the cutoff, by adding specific circRNAs manually or even selecting a specific subset of circRNAs to check (Fig. 1C).

### Setting a cut off in the RNaseR/mock ratio

A given circRNA would be consider real if the ratio of the signals obtained between the RNaseR and mock samples is higher than a given threshold/cutoff value. The choice of this threshold is somehow arbitrary, and it is initially based on the distributions of these ratios for the circRNA candidates and their hosting mRNAs in a given tissue/species. Moreover, this threshold can be accommodated by the user at a later point. Previous work showed that circRNA junctions found by more pipelines are more likely to be generated from bona fide circRNAs [23, 35]. Therefore, we decided to use that criterion to guide the selection of the most appropriate cutoff for the RNaseR/mock RNAseq ratio. Briefly, we looked at the proportion of validated and false-positive circRNAs when circRNAs were identified by one to five pipelines. As we wanted to choose a reasonable criterion for distinguishing bona fide from false-positive circRNAs, we looked at this parameter as we changed the cutoff value (see Table S1, Fig. 2C, D, and Additional file 1: Figure S1A). We observed that using a cutoff between 0.85 and 0.95 (a cutoff that assumes 5–15% of linear mRNAs have some resistance to RNaseR) we included most circRNAs identified by all pipelines (while eliminating 85–95% of linear RNAs, see Additional file 2: Table S1, Fig. 2C, D). This was also the trend when we examined circRNAs detected by individual pipelines (Additional file 1: Figure S1B). For cutoffs over 0.95, the proportion of the true circRNAs selected decreased rapidly even for those circRNAs detected by all the pipelines, which are highly likely to be real (Fig. 2C, D, and Additional file 1: Figure S1A). On the other hand, higher error rates result in the inclusion of false positives (i.e., as the cutoff was made less stringent; Fig. 2C and Additional file 1: Figure S1A). To determine the exact cutoff, we assumed that circRNAs detected by multiple pipelines that have medium to high levels of expression are more likely to be real. For instance, a cut off of 0.95 still eliminates some circRNAs that are of medium expression and detected by multiple pipelines (see comparisons of distribution of expression of true and false circRNAs for different thresholds in Additional file 1: Figure S1C). Therefore, for this particular dataset, we chose to set up the cutoff at 0.90. At this cutoff, the large majority of the circRNAs with middle or high expression and which are detected by multiple pipelines are identified as true (Fig. 2E top), with a significantly higher RNaseR/mock sensitivity (Fig. 2E bottom). Importantly, most of the circRNAs with medium/high expression which we catalog as false are pipeline specific (Additional file 1: Figure S1D). In sum, using both the RNaseR sensitivity and the expression criteria, we likely included most real circRNAs, while minimizing the number of false positives. It is important to point out that the cutoff-derived percentage of potential false positives is purely theoretical and it is likely below this 10% for two different reasons: (1) As previously described [40], a subset of linear RNAs is resistant to RNaseR, possibly due to secondary structures; (2) The estimated 10% is based on measurement of junctions of mRNAs that host circRNAs, some of these junctions indeed could also be shared with other circRNA isoforms making the number of linear molecules less than the estimated 10%.

By utilizing the SCRP strategy with a cutoff of 0.9, the proportion of circRNAs identified by all pipelines increased from about 35% to almost 60% (Additional file 1: Figure S1A). Importantly, some circRNAs detected by only one or two pipelines were identified as true circRNAs by the SCRP approach, demonstrating the utility of running multiple pipelines in the annotation step. However, most of the pipeline-specific circRNAs have an RNaseR/mock ratio lower than the cutoff value (see dotted line in Fig. 2C, Additional file 1: S2E, and Additional file 2: Table S1) indicating that they are likely false positives and rightly eliminated by the annotation step of SRCP. One of the main risks/possible drawbacks of our approach is to exclude bona fide circRNAs due to a too stringent cutoff (false negatives). In any case, more circRNAs can be manually added to the “true” list if there is additional evidence of their circularity or if the researcher is particularly interested on them. Using the chosen threshold, we determined how well each pipeline annotates circRNAs. The proportion of true positives is variable between the pipelines as well as the percentages of false positives (Fig. 2F). These results demonstrate that the utilization of multiple pipelines and analysis of RNaseR susceptibility, as implemented in SCRP, is more comprehensive than any previously described pipeline for annotating circRNAs.

### Expression and genomic features of bona fide circRNAs

Given our identification of a set of bona fide circRNAs, we asked whether these circRNAs could be identified using other genomic and/or expression features. As circRNAs lack a polyA tail, their appearance in polyA^+^ libraries is usually an indicator of a false positive (the exceptions are the few circRNAs that contain stretches of adenosine within them). Indeed, validated circRNAs had very low expression levels in polyA^+^-selected RNA-seq libraries (Additional file 1: Figure S2A). In addition, bona fide circRNAs tend to be expressed at higher levels and be longer than false-positive circRNAs identified in total RNA-seq libraries (Additional file 1: Figures S2B and S2C). These two features could potentially be useful in identifying circRNAs.

To identify additional genomic feature differences between circRNAs and linear transcripts, we compared a list of circRNAs to a group of transcripts containing exons randomly selected from the group of exons that do not form circRNAs (see “Methods”). Neither intron length nor GC content clearly discriminated between true and false-positive circRNA junctions, although exons included within true- and false-positive circRNA junctions were flanked by much longer introns than randomly selected exons (Additional file 1: Figure S2D), in agreement with previous analysis [42]. In addition, the exons on both sides of bona fide circRNAs tended to be annotated (Additional file 1: Figure S2E). Hence, many of the junctions that are wrongly classified as circRNA-specific are generated from poorly annotated genes. Interestingly, bona fide circRNAs tended to be hosted by genes with more exons than false-positive circRNAs or randomly selected exons (Additional file 1: Figure S2F). As previously described [43], bona fide circRNAs were more likely to be generated from the second exon of the hosting gene than were randomly selected exons, although we observed a similar trend for the false-positive candidates (Additional file 1: Figure S2G). Thus, bona fide circRNAs have genomic features that distinguish them from exons that are not circularized, but these differences are not enough to design a non-experimental criterion for the identification of “true” circRNAs from RNA-seq data.

### Annotated circRNAs can be accurately quantified using SRCP

circRNA pipelines identify different sets of circular junctions. As these pipelines utilize different quantification approaches, their results cannot be merged and compared for downstream analysis. The methodology described here overcomes this limitation. SRCP merges potential circRNA junctions identified using various pipelines and then quantifies them using a seed-matching approach. In this approach, we require that a seed of certain size around the circular junction must be included in the RNAseq in order to quantify it as a circular read. This allows calculation of differential expression of all validated bona fide circRNAs, independently of which circRNA pipelines detected them.

Theoretically, the SRCP performance should be strongly affected by the length of the seed utilized to identify the circRNA junctions. Longer seeds should unequivocally identify the circRNA junction, and shorter seeds should result in higher false-positive rates. We ran the SRCP with three different seed lengths (4, 6, and 10 bases) using one read of the paired-end RNAseq sample (SRR1197359) which we computationally downsized in length and depth. As expected for each library depth, we detected more circular reads and a larger repertoire of circRNAs as we shortened the seed (Additional file 1: Figure S3A). Changing the seed length while keeping the read length and library depth constant only marginally altered the circRNA detection, although seed length was more important for shorter RNA-seq reads than longer (Additional file 1: Figure S3B). For further experiments, we utilized a seed of 5 bases from each side of the backsplicing junction (10 bases total when aligning to the backsplicing junction).

To determine the accuracy and sensitivity of our seed-based quantification, we used both simulated and experimental data. First, we simulated seven sets of RNA-seq reads based on known circRNA junctions from *Drosophila melanogaster* (see “Methods”). Each set was designed to contain half of the number of circRNA read counts as the one before (Additional file 2: Table S2). Importantly, these simulated data utilized circRNA junctions that were identified by all pipelines, and hence the results reflect only the quantification aspects of the pipelines. Of the pipelines tested SRCP, CIRI, circExplorer, and acfs detected the most circRNA reads in each sample with very small variations, while find_circ and circRNA_finder were less sensitive (Additional file 1: Figure S4A). This means that SRCP, CIRI, circExplorer, and acfs are sensitive enough to detect low-abundance circRNAs represented by only 10 to 40 reads per circle per sample (Additional file 2:Table S2, samples 5-7). When we evaluated the number of circRNA reads detected for the 100 most highly expressed circRNAs, we observed similar performances for SRCP, CIRI, circExplorer, and acfs. These pipelines identified significantly more junctions than find_circ and circRNA_finder (Additional file 1: Figure S4B). These results demonstrate that SRCP sensitivity is comparable to the most sensitive available circRNA-detection pipelines. However, these conclusions derived from simulated single-end data and do not contain the variety of reads and signals existent on in vivo data.

**Fig. 3:**
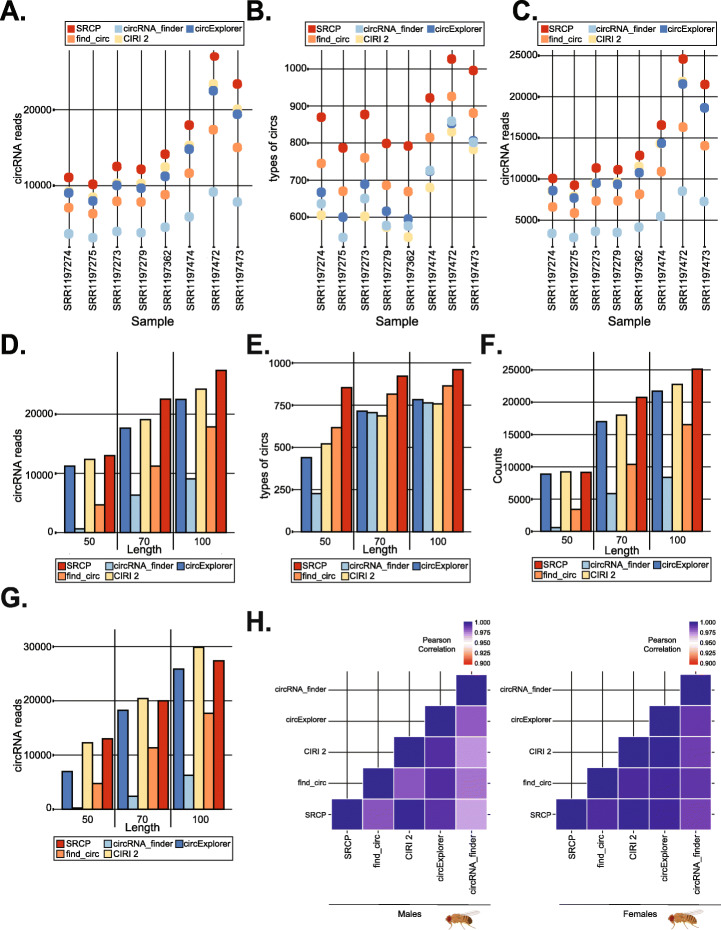
circRNAs can be accurately quantified using seed matching. **A** Total number of circRNA RNAseq reads for True circRNAs detected by the different pipelines in the 8 samples from female flies of the SRP001696 dataset. **B** Number of types of true circRNAs identified by each pipeline in each sample, using the same dataset as in (**A**). **C** Number of true-common circRNAs identified by each pipeline in each one of the indicated samples. As stated in the text, true-common circRNAs refers to circRNA identified by all the pipelines in the mock samples and with a RNaseR/mock ratio above the cutoff value. **D** Total number of circRNA RNAseq reads for true circRNAs detected by the different pipelines in one of the samples (SRR1197359) in the intact PE reads (100 bases long) or after computationally truncate them to 50 or 70 bases long. **E** Number of types of true circRNAs identified by the different pipelines in the SRR1197359 sample in the whole or truncated (to 50 or 70 bases) reads. **F** As in **D** and **E**, showing the number of reads originated from the True-common circRNAs. **G** Total number of true circRNA RNAseq reads for the different pipelines when analyzing the two reads (R1 and R2) of the SRR1197359 sample independently (as single-end reads). As in (**D–F**), we have done the analysis in the whole read (100 bases) or after it was truncated to be 50 or 70 bases long. **H** Pearson correlation heatmap visualizes quantification by the different pipelines and SRCP in the male and female fly samples (SRR1197359 and SRR1197473 respectively)

To further compare SRCP with the other circRNA computational pipelines, we utilized a previously published dataset of fly heads at different ages [44]. Interestingly, SRCP has the highest quantification power among the tested pipelines, as it was able to detect a significantly larger number of circRNA reads from all the samples (Fig. 3A). This is not due to the larger number of circRNAs identified by SRCP (Fig. 3B), as it also detects more circRNA reads while quantifying only the set of circRNAs detected by all pipelines (true circRNAs, which are identified and quantified by all the pipelines, Fig. 3C). These results strongly suggest that the use of SRCP lowers the chance for false positives with higher sensitivity than the more sensitive currently available circRNA-detection pipelines.

As most pipelines rely on the identification of hybrid RNA-seq reads that align to two regions of the transcriptome, circRNA identification and quantification becomes less efficient as RNA-seq reads become shorter. In theory, SRCP should not have this problem as once indexes are established, the quantification of circRNAs relies on only a certain number of the seed bases to align perfectly (the seed length can be adjusted by the user but the default is 5 from each side). To test this hypothesis, we compared the performance of SRCP with the different pipelines in two of the samples in which the reads were maintained 100 bases long or computationally truncated using fastx trimmer to 50 or 70 bases. SRCP consistently outperformed the other pipelines at all the assayed lengths (Fig. 3D and Additional file 1: Figure S4C). As expected, SRCP also detected more validated/true circRNAs at the three lengths of the reads tested (Fig. 3E and Additional file 1: Figure S4D). To solely compare the quantification power of SRCP with the other pipelines, we restricted the analysis to those bona fide (“true”) circRNAs that are detected by all pipelines (common-true circRNA set). Also, in this subset, SRCP outperforms the other pipelines while the subsamples are of different length (Fig. 3F and Additional file 1: Figure S4E), except for 50 bases long reads, in which CIRI and SRCP perform similarly. These results suggest that SRCP could be also used to quantify circRNAs from short RNA-seq libraries, like those generated from RNA precipitation-based methods such as RIP and CLIP. Only SRCP and CIRI will be effective with techniques that generate even shorter RNA-seq reads like ribosome foot printing, for which we utilized a similar approach in the past [16].

Then, we determined the quantification power of the different pipelines when utilizing single-end (SE) rather than paired end (PE) RNAseq. To do so, we utilized the same dataset but ran the pipelines utilizing the data as single end reads. We compared their power to detect circRNAs from the intact or computationally truncated reads and determined how they compare with the PE assessments. Interestingly, while utilizing SE reads, the number of total (R1 + R2) circRNA reads identified by CIRI2 is slightly higher than SRCP (Fig. 3G and Additional file 1: Figure S4F). This is not surprising as CIRI2 corrects for false positive RNAseq reads as well as for potential double counts when using PE data [45]. Therefore, CIRI2 might identify false-positive reads while utilizing single-end reads, which are properly eliminated if PE data is available. None of these is an issue for SRCP, as it only identifies reads aligned to the correct strand and eliminates any RNAseq reads with strong alignment to the transcriptome and/or genome (Fig. 1D). Moreover, careful examination of the reads determined that no PE read is counted twice by SRCP in this dataset. In conclusion, these data demonstrate that SRCP outperforms all the other tested pipelines with PE. However, CIRI2 performs particularly well in single-end (SE) data (Fig. 3G), although it detects a lower number of circRNAs types and junctions when the analysis is not restricted to the circRNAs detected by all the pipelines (common circRNAs).

We then compared the quantification values obtained by the different pipelines and observed a strong correlation between them in both males and female samples (Fig. 3H). To further asses the quantification power of SRCP, we calculated the correlation between replicas for the different pipelines. In this analysis, we also included sailfish-circ [46]. This pipeline utilizes all the RNAseq reads (not just the backsplicing junction reads) and requires the previous annotation of circRNAs (we utilized the same list of “true” validated circRNAs used while quantifying with SRCP). Most pipelines displayed very high correlation between biological replicas (Pearson correlation > 0.98) with SRCP having the highest correlation values for most samples (Additional file 3). Sailfish-circ correlations were consistently lower, although still acceptable.

### SRCP identifies more differentially expressed circRNAs than other individual pipelines

**Fig. 4:**
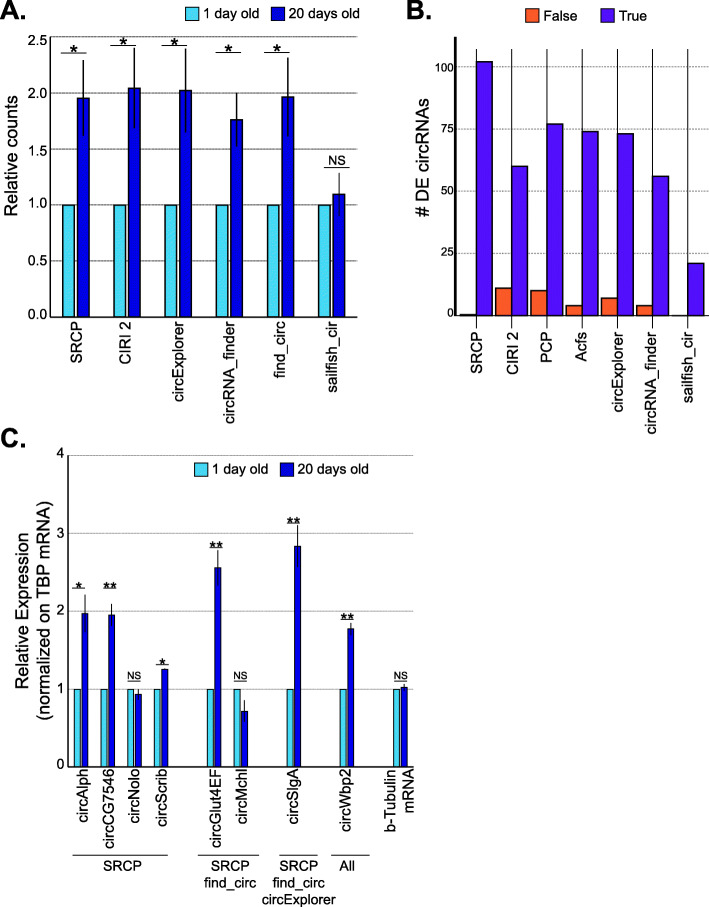
SRCP enables accurate identification of more differentially expressed circRNAs than other pipelines. **A** Relative amount of total circRNAs reads (for true circRNAs) in young (1 day old) and aged (20 days old) flies. The total number of circRNAs was calculated using the individual pipelines, and reads assigned to true circRNAs were added up for each condition. The average of young flies was normalized to 1. * Indicates significance (*t*-test, *p* value < 0.05), while NS indicates no statistically significant differences. The error bars represent the standard error of the mean (SEM). **B** Number of true (violet bars) and false-positive (orange bars) differentially expressed circRNA found by SRCP and the other circRNA-identification pipelines. **C** Validation of DE circRNAs by qPCR. Expression of target circRNAs and beta-Tubulin mRNA were normalized on the level of TBP mRNA. We then plotted the average of 3 independent biological replicates and the error bar represents the SEM. The average of young flies (1 day old) was normalized to 1. We performed *t*-test to compare 1 day old vs 20 days old. * Indicates *p* value < 0.05, ** *p* value < 0.005, NS non-significant

To benchmark SRCP, we utilized a dataset that has been used previously to show that the global levels of circRNAs increase with age [32]. We then compared the total number of circRNA reads in young (1 day old) and aged (20 days old) flies utilizing the different pipelines. In agreement with previous findings, most pipelines (including SRCP) found a strong increase in the total circRNA counts at day 20 in comparison to day 1 (Fig. 4A). Surprisingly, sailfish-circ fail to show that increase (Fig. 4A).

We then used DESeq2 [47] and looked for differentially expressed circRNAs between young and aged flies. We found that the SRCP pipeline more effectively detected differentially expressed bona fide (true) circRNAs than all other pipelines (Fig. 4B and Additional file 2: Table S3). Using the SRCP approach, we identified 102 bona fide circRNAs that are differentially expressed (FDR < 0.05) between these two conditions; all the other pipelines identified less than 80 (Fig. 4B). Importantly, using each of the pipelines without pre-filtering for true-positive junctions resulted in sets that included many false positives (Fig. 4B and Additional file 2:Table S3). These results emphasize the importance of the annotation step that we present here. Using our annotation procedure lowers the number of false positives and thus results in higher accuracy in further analyses.

We then utilized RT-qPCR to validate a subset of the circRNAs that were detected as differentially expressed (DE) by SRCP between young and aged flies. For the validation, we chose one circRNA (circWbp2) that we found DE by all pipelines, four that were found only by SRCP (circAlph, circCG7546, circNolo, and circScrib), and three that were found to be DE by SRCP and one or two additional pipelines (circGlut4EF and circMchl, and circSlgA, respectively). As expected, the levels of the control mRNA (*b-tubulin*) were similar in young and aged flies (Fig. 4C). Importantly, 6 out of the 8 tested circRNAs increased their levels significantly with age. Among them, 3 were out of the 4 found DE only by SRCP (Fig. 4C), demonstrating that the changes (that could only be detected by SRCP) are real. As circNolo and circMchl were barely detected by qPCR (with Cqs beyond cycle 30), it is not possible to completely rule out that these circRNAs also increase with age. In sum, these results show that the use of our two-step procedure extracts more accurate information from the RNA-seq data than other circRNA identification pipelines, although the computational prediction is still not perfect, since some data could not be validated experimentally.

### Building a mammalian database of validated circRNAs for universal SRCP use

**Fig. 5:**
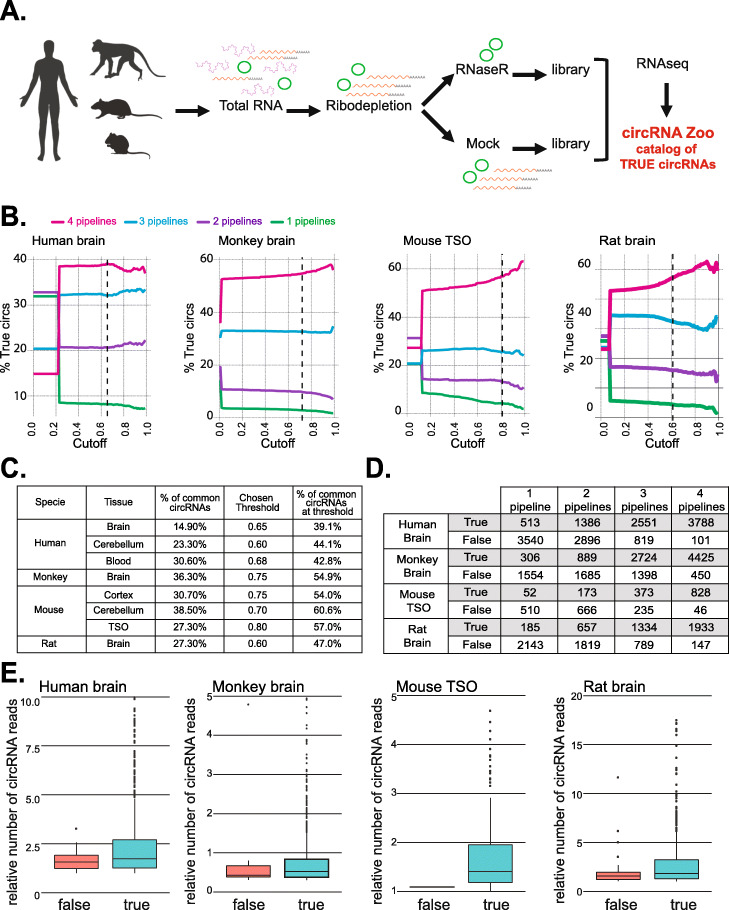
Validation of bona fide circRNAs in four mammalian species. **A** Strategy utilized to identify bona fide (true) circRNAs from several tissues from four different mammal species. **B** The percent of circRNAs identified as “true” positives as a function of the cutoff for circRNAs identified by 1, 2, 3, or 4 of the pipelines used in the indicated species and tissue. **C** Table summarizing the percentage of common circRNAs selected as true at the chosen threshold. **D** Table summarizing the number of pipelines that identify the sets of true and false circRNAs identified from the indicated tissues and species. For building this table, we utilized the thresholds marked in (**B**) as a dotted line and indicated in the table in (**C**). **E** Boxplots showing the distribution of expression of the true and false circRNA that are identified in the indicated species

To extend the use of SRCP to mammals, we generated and sequenced RNAseq libraries from mock and RNaseR-treated RNA obtained from mouse, rat, monkey, and human samples (Fig. 5A). In the case of human and mice, we utilized different tissues and brain regions (see Additional file 2: Table S4). Overall, we sequenced almost 2 billion 150-base-long pair-ended (PE) RNAseq reads (Additional file 2: Table S4).

To identify circRNAs from those samples, we utilized four different pipelines (CIRI2, find_circ, circRNA finder, and circ_explorer), generated the circRNA index as we did for the *Drosophila* samples (see above) and run SRCP to identify the circRNA and linear junctions for each tissue/specie. We then proceeded to determine the abundance of all circRNAs in the RNaseR-treated and mock samples and plot them according their RNaseR resistance. As for *D. melanogaster*, we observed that circRNAs presented higher RNaseR/mock ratios than their linear counterparts (Additional file 1: Figure S5). However, the shape and value of the distributions of this ratio between linear and circRNAs differ between samples, likely due to different efficiencies of the RNaseR treatment or maybe the tissue and origin (Additional file 1: Figure S5). These observations prompt us to determine specific thresholds for each organism/sample tissue. For most samples, we detected less than 30% overlap between the circRNAs detected by the different pipelines (see magenta line at the y-intercept in Fig. 5B and Additional file 1: Figure S6A, and percentage of common circRNAs in Fig. 5C). To choose the threshold, we assumed that circRNAs detected by all the pipelines are more likely to be bona fide circRNAs. In general, these circRNAs presented much higher resistance to RNaseR and hence their relative abundance tended to increase as we increased the cutoff (Fig. 5B, Additional file 1: Figures S6A and S6B). Last, when possible, we adjusted the threshold to include circRNAs detected by several pipelines and that are expressed at medium or high levels. Based on these criteria, we selected specific thresholds and defined the lists of “true” circRNAs for the different species and tissues. Indeed, we observed that the percentage of common circRNAs was significantly increased at the selected threshold compared to before setting this cutoff (Fig. 5C, Additional file 1: Figures S6A and S6B). In addition, we observed that some circRNAs were resistant to the RNaseR treatment in one tissue but fall just behind the threshold in a different one. This could be due to a given junction being originated from a bona fide circRNA in one tissue and from spurious splicing product in other, or just to different efficiency of the RNaseR treatment, low coverage, or other technical artifacts. We favor the latter explanation based on the current knowledge in the field, as well as on the finding that most of these circRNAs were lowly expressed in the tissue in which they were found to be in the “false” group. Therefore, we expanded the list of true circRNAs by requiring their RNaseR/mock ratio to be over the threshold just in one tissue. This not only might account for different abundance in various tissues and efficiency of the RNaseR treatment among samples, but also as a biological replicate. To further diminish the possibility of false negatives, we performed a similar analysis in a recently published mock/RNaseR dataset from human, mouse, and monkey brains [48]. Indeed, very few circRNAs detected in the second study as “true” were qualified as “false” in our previous analysis. We then incorporated these circRNAs, which served as biological replicas for our RNaseR-treated samples. Indeed, by collapsing tissues and incorporating these biological replicas, only a small fraction of the false circRNAs were detected by all the pipelines (Fig. 5D and Additional file 1: Figure S6C). Moreover, we observed that there was almost no “false” common circRNAs with middle or high expression level (Fig. 5E and Additional file 1: Figure S7A), validating the additional criteria we utilized. Indeed, the few circRNAs that were considered “false” and still have middle expression in the mock samples, are clearly degraded by the RNaseR treatment (Additional file 1: Figure S7B). We created then the list of true circRNAs (Additional file 4). To compare this informed cutoff criteria with a more standardized one, we tested what happened in some of the samples if we follow the approach utilized by [23, 35] in which circRNAs were considered true when they were enriched more than 5-fold in the RNaseR-treated samples. While this cutoff criterion is reasonable and can work very well in particular samples, it does not take into account the variability of the efficiency of the RNAse treatment between samples and differences in sequencing depth. Indeed, the specific enrichment of the circRNAs upon RNaseR treatment can vary greatly between samples, although linear mRNAs are always clearly more sensitive to RNaseR (Additional file 1: Figure S5). Therefore, a predetermined cutoff might lead to the exclusion of many bona fide circRNAs (the red dotted lines in Additional file 1: Figure S5 indicate the position of the cutoff if a 5-fold enrichment is utilized as criteria for dividing true from false circRNAs). Although using this pre-set cutoff did not make much difference in the mouse cerebellum sample, the outcome was completely different in the rest of the samples, in which this criterion is too stringent and leads to the elimination of thousands of circRNAs that were identified by multiple pipelines (Additional file 2: Table S5). Specifically, for most of the samples the 5-fold criteria would be comparable to use a cutoff of 0.95 or higher. Therefore, we concluded that using an informed a posteriori approach to pick the exact cutoff is safer than using a predetermined cutoff solely based on RNaseR/mock fold enrichment and leads to less false negatives. Hence, following these experiments and the creation of the list of true circRNAs, SRCP can be used in any of these species to accurately detect and quantify circRNAs. It is important to point out that this list can be easily modified when: (1) more datasets are available for one specie/tissue; (2) if some circRNAs are validated by independent methods; (3) any of the circRNAs in the “true” list is shown to be an artifact.

### The miRNA effector protein AGO2 binds a subset of circRNAs in the human brain

**Fig. 6:**
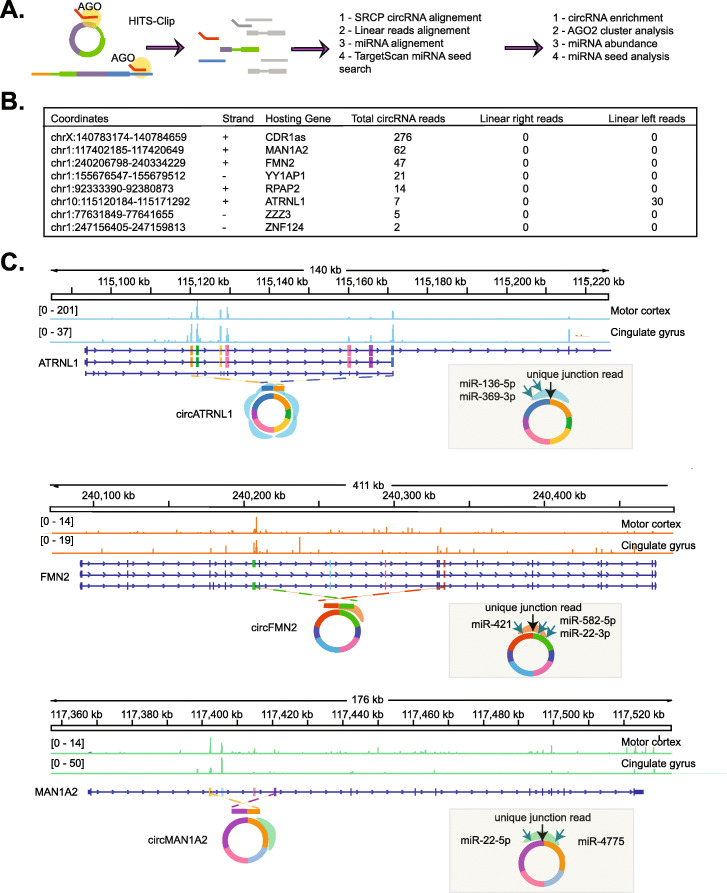
circRNAs bind to AGO2 in the human brain. **A** Scheme of the approach utilized to analyze the AGO2 HITS-CLIP data set. **B** Table summarizing the circRNAs for which SRCP identified backsplicing reads in the AGO2 CLIP data. The list contains circRNAs that were found in at least 2 different human brain samples. Linear “left” or “right” reads refers to junctions encompassing the more proximal or more distal exon within the circRNA with the exon before or after in the linear mRNA respectively. **C** IGV snapshot of the AGO2-CLIP raw data in the region containing the gene hosting the AGO2-bound. We marked the backsplicing junction with a dashed line. We represented with a colored shadow the AGO2 cluster enrichment analysis and indicated the miRNAs for which an overlapping miRNA seed was identified

Previous work has shown that at least two circRNAs (CDR1as and circSry) strongly bind to the miRNA-effector protein AGO2 and that this binding might result on the regulation of specific miRNAs (mir-7 for CDR1as [11, 12] and miR138 for circSry [12]). Since then, many studies have proposed the potential function of circRNAs as miRNA sponges, but until now, there is no clear evidence of a general sponging activity by circRNAs. To find circRNAs that interact and potentially regulate miRNAs or are regulated by miRNAs, we utilized SRCP to identify circRNA junctions from previously published AGO2-HITS-CLIP data from two regions of the human brain [49]. CLIP data is particularly challenging for many circRNA-identifying algorithms (like find_circ) because the library inserts are usually small (50–70 bases, indeed this is the case for the analyzed dataset, see Additional file 1: Figure S8A). When we applied SRCP in these datasets, we found 8 circRNAs with backsplicing reads in more than one sample (Fig. 6A, B). As expected, the circRNA with more AGO2-CLIP reads was CDR1as which is highly expressed and contains 71 miR-7 binding sites and which has been shown to strongly bind AGO2 in vivo [18]. Actually, we found up to 3 of the 71 miR-7a sites in the reads spanning the backsplice junctions of CDR1as.

To provide additional evidence of the binding of these circRNAs to AGO2, we extracted the reads supporting the backsplicing junctions and computationally predicted miRNA sites using TargetScan [50]. We filtered the candidate miRNAs using expression data from the same report (by sub-setting the top 15% expressed miRNAs, Additional file 1: Figure S8B and Additional file 5) [49]. This allowed us to restrict our list to a few miRNA candidates per circRNA (Additional File 5, Fig. 6C, and Additional file 1: Figure S8C).

Interestingly, for almost all the AGO2-bound circRNAs, we could not find the corresponding linear junction reads (Fig. 6B). This is not surprising, as circRNAs are generally produced from exons at the beginning or middle of the gene and the binding of miRNAs (and hence AGO2) to mRNAs happens predominantly on the 3′UTR. The exception to this is ATRNL1 gene, which has also reported peaks in exons that are not part of the circRNA. In fact, circATRNL1 is the only circRNA in which we could detect the linear counterpart splice junction (Fig. 6B and Additional file 5). However, ATRNL1 generate additional circRNAs, as stated in circBase, which could explain the additional peak and the presence of linear CLIP reads in the middle of the RNA. In sum, these results demonstrate that in the human brain only a handful of circRNAs are strongly bound to AGO2 and could potentially regulate the function of specific miRNAs.

## Discussion

In this study, we present a novel computational pipeline that facilitates accurate annotation and quantification of circRNAs from RNA-seq data. The method, which we call Short Read circRNA Pipeline (SRCP), quantifies circRNAs with high sensitivity and with a low number of false negatives. This method is general and is not limited to a specific circRNA detection pipelines. The quantification step is simpler than all other pipelines since it does not search de novo for circular RNA junctions.

A number of circRNA identification and quantification tools have been described including acfs [26], DCC [27], segemehl [28], CIRCexplorer [29], KNIFE [30], MapSplice2 [31], circRNA_finder [32], CIRI [33], and find_circ [11]. All can be used to identify and quantify circRNAs, but none of them alone identify all circRNAs. Indeed, only about 30% of circRNAs were identified by all the tested pipelines. Therefore, when looking for differentially expressed circRNAs, researchers generally either chose one pipeline or focus on those circRNAs detected by multiple pipelines. This is unfortunate as this procedure eliminates many real circRNAs from the analysis. Moreover, as different pipelines utilize different quantification approaches, their results cannot be combined. Besides, an important number of the potential circRNA reads, particularly the ones detected only by one pipeline, are not originated from real circular reads. This is problematic for two different reasons. First, it might mislead the researcher into studying a molecule that is not real. Second, it complicates the statistical analysis by increasing the number of tests to be performed and diminishing the significance of the results. SRCP solves both these issues by first annotating bona fide circRNAs and then utilizing common criteria for quantification. Thus, our approach analyzes a much larger group of circRNAs than would be interrogated using only one method or the overlapping circRNAs among several methods and accurately quantifies circRNAs minimizing the amount of false positives and negatives. The strategy utilized by SRCP is somehow similar to the one used by KNIFE [30]. However, KNIFE examines and counts all possible splicing junctions, making it slower and very demanding from the computational point of view.

Another strength of our approach is that the user can modify the annotation of the false-positive circRNAs identified by different circRNA detection pipelines. As the annotation is based on the RNaseR-treated library to the total (rRNA depleted) library (referred as mock) ratio, the user can control the false-positive rate. While the RNaseR/mock ratio is the gold standard for validating the circularity of RNA molecules, some linear highly structured RNAs are also resistant to this RNAse [40]. In principle, this is not problematic unless any of these RnaseR-resistant mRNA presents an artifactual backsplicing junction. Nevertheless, accurate annotation of circRNAs is challenging due to difficulty in assigning the cutoff, which might vary from sample to sample depending on the tissue, organism, and efficiency of the RNaseR treatment. Here, we assumed that false-positive circRNAs are as sensitive to RNaseR as linear RNAs and that circRNAs identified by several pipelines tend to be bona fide circles. However, additional criteria could be utilized to determine the threshold in a more informed manner. For example, in pair-ended data, the reads do not map to the backsplicing junction could be potentially utilized to determine if the library fragment is contained within a circRNA or not. In addition, circRNAs that have been validated by an independent method, in another tissue, or in a separate experiment can be incorporated into the list at any time. As for most of the other available pipelines, one limitation of SRCP is the identification of alternative circRNA isoforms, especially those that involve the presence or absence of exons within the circRNA and the same backsplicing junction. In these cases, all circRNA identification and quantification pipelines, including SRCP, will quantify the sum of all the different isoforms. Therefore, the detection and quantification of those isoforms would require a different type of experimental procedure, like the ones recently described utilizing full sequencing of circRNAs [51, 52] or a computational approach, as described for CIRIFULL [53].

Interestingly, in our analysis of *Drosophila* circRNAs identified and quantified using SRCP, we found that bona fide circRNAs share some genomic features and expression characteristics. These include their very low abundance in polyA-selected RNA-seq libraries, slightly lower expression levels, larger exon size, hosted by genes with more introns, and better genomic annotation compared to randomly selected exons. Although none of these features individually allowed us to computationally discriminate between “true” and “false” circRNAs, machine learning approaches might be trained to take these features into account to improve de novo identification of circRNAs to diminish false negatives.

Importantly, SRCP requires the presence of a validated list of circRNAs which can only be generated by running several pipelines in mock and RNaseR-treated samples. Here, we provide a comprehensive list of validated circRNAs for human brain and blood as well as for mouse, rat, and monkey brain. Nevertheless, new datasets will have to be generated and analyzed for using SRCP in other tissues and/or organisms. Once generated, those lists can be utilized in further experiments and the computational time and framework for analyzing circRNA expression will diminish considerably.

## Conclusions

In sum, SRCP is a novel method that combines circRNA annotation and an efficient algorithm for their quantification. To identify false positives from the several different circRNA-identification pipelines, we compared the expression of the putative circRNAs in mock and RNaseR-treated samples. We adjusted the threshold/cutoff of the RNaseR/mock ratio for minimizing the number of false negatives by taking into consideration the detectability by several pipelines and the expression level of the circRNA. By comparing the results obtained using SRCP with the ones obtained using five circRNA identification and quantification pipelines on multiple simulated and real RNA-seq datasets, we found that SRCP identifies more differentially expressed circRNAs than any of the other methods. Finally, SRCP allows quantification of circRNAs that were identified by different pipelines.

## Methods

### Creation of a circular reference

Using available genome annotation, we extract for every putative circRNA all its potential transcripts. We do this using bedtools intersect, we intersect the circRNA coordinates with the genome transcriptome annotation. Next, we score the transcripts as follows: (i) If the start coordinate and the end coordinate of the circle are both exactly on a 5′ and 3′ boundaries of the transcript’s exons, the score is maximal. (ii) If only one coordinate is exactly on an exon boundary and the other is not, the score is 1. (iii) If neither coordinate is on any exon boundary, the score is 0. Next, we choose for each circRNA in the database, a transcript that best fits the circle. This is the transcript with the highest score or, if a few transcripts all have the same highest score, one is randomly chosen. Next, using bedtools getfasta [54], we extract the circle sequences of the chosen transcript or transcripts and build an index using bowtie2 build [55]. This is done for each potential circRNA.

### Creation of a linear index

To create linear references, for each circRNA junction, we select the closest annotated exons upstream and downstream of the circular junction. We then extract the FASTA sequences for those exons using bedtools get_fasta [54]. We concatenate the upstream exon sequence to the circular sequence, we name this “linear_left.” We also concatenate the downstream exon sequence to the circRNA sequence this is “linear_right”. We then build its corresponding index using bowtie2 build for the right and for the linear_right and linear_left FASTA sequences [55].

### Detection and quantification of circRNAs

RNA-seq reads are aligned to the circular index with bowtie2 [55]. The reads that align to the circular index are next aligned to the genome and to the transcriptome. The alignment to the genome is done to remove reads that come from unannotated genes. The alignment to the transcriptome is done to ensure that no reads that are originally from a linear transcript are included in the detection and quantification of circRNAs analysis. Reads that align to the circular index and not to the genome or transcriptome are candidates for circular circRNA reads. To be confirmed as a circRNA, the junction must be included in the read and a certain number (*j*) bases upstream or downstream or the putative junction have to match the exon with no mismatches.

To quantify circular reads, the number of reads that align to the circular index for each circRNA is calculated. For linear reads, the number of reads that align to the linear index is calculated for all transcripts once for the downstream of the circle and once to the upstream side of the circle.

### Selection of the cutoff value

To select an appropriate cutoff value, we compared the number of circular and linear RNA reads classified as RnaseR resistant for an array of different cutoff values of the RNaseR/mock ratio (Additional file 1: Figure S2A, B). We performed this analysis for all the pipelines utilized in this study including SRCP. We first re-scaled the data. The cutoff value is expressed as the percentage of linear RNAs would be considered RnaseR sensitive (i.e., a 0.90 cutoff value excludes 90% of the linear mRNAs). A cutoff of 95% removes most linear RNA reads (Fig. 2E). Interestingly, circRNAs identified by more than one pipeline tended to be enriched among those considered real at higher cutoff values (Fig. 2E). For example, a cutoff of 0.8, 98% of the circRNAs that were found by all pipelines (and are believed to be true circRNAs) were annotated as bona fide circRNAs. On the other hand, at a cutoff of 0.99 only 77% were found by all five pipelines to be bona fide circRNAs. At a cutoff of 0.95, 92% were bona fide circRNAs (Fig. 2E, Additional file 2: Table S1). Thus, the lower the cutoff, the more false positives we introduce. Elevating the cutoff results in fewer false positives but also fewer true positives.

### Collapsing tissues from the same species

In some scenarios, a circRNA can pass the cutoff in one sample and not in another, for example, if we have big differences in coverage of the mock and/or if a circRNA is expressed in certain condition that was present in one of the tissues and not in another. To try and minimize the false-negative detection due to these different reasons, we decided to rescue by looking at the collection of all collapsed “true” circRNAs from the same species. For species which we had more than one tissue, such as human and mouse, we first calculated the RNaseR/mock ratios and the cutoffs. We then annotated the circRNAs accordingly.

Next, we collected all the “true” circs from each of the tissues (of the same species) and used that as a reference to exclude “false” circRNAs. If a “false” circRNA was found in the collapsed “true” circ list, we changed its annotation to “true.” The final “true” circRNA list per tissue consists of circRNAs that passed the cutoff plus some circRNAs that passed the cutoff in a different tissue.

### Creation of simulated data

In order to create a simulated dataset, we took all the circular junctions found in *Drosophila melanogaster* heads and extracted the fasta sequences for the junction area (70 bases upstream and downstream of the circular junction) plus the fasta sequences for the upstream and downstream exons. We then randomly selected start positions on the circRNA and generated 70-base circular reads (from the circle sequences) and linear reads from the upstream and downstream exons.

### Selection of random exons

To select random exons, we used the *Drosophila melanogaster* annotation from UCSC. For each gene, we selected the starting exon in the transcript. We then randomly selected the desired number of exons. Using the gene annotation from UCSC, we extracted the annotation for each of these random made transcripts. We then use bedtools getfasta to extract the corresponding fasta sequences for each of these random transcripts.

### Detection and quantification of circRNAs with the different pipelines

We ran circRNA detection pipelines Acfs, CircExplorer, circRNA_finder, CIRI2, and find_circ with default parameters with the dm3 genome and genome annotation from the UCSC genome browser as input. In all cases, we utilized the default parameters.

### Detection and quantification of circRNAs with the different pipelines for additional human, mouse, and monkey datasets

We downloaded the data ran SRCP on these data, using the circ lists we had established from the previous analysis of the atlas. We used the same parameters as we used for the datasets in the atlas with hg38(human), mm10(mouse), and rheMac10(monkey) genomes. The corresponding genome annotations were downloaded from the UCSC genome browser and used as input. In all cases, we utilized the default parameters.

Similar to the rescue we performed by collapsing the tissues, we collected the “true circRNAs” found in these datasets while in our data did not pass the cutoff and changed their annotation to “true.”

### Detection and quantification of circRNAs with the different pipelines for species data

We ran circRNA detection pipelines CircExplorer, circRNA_finder, CIRI2, and find_circ with default parameters with hg38(human), mm10(mouse), rheMac10(monkey), and rn5(rat) genomes. The corresponding genome annotations were downloaded from the UCSC genome browser and used as input. In all cases, we utilized the default parameters.

### Examination of PE reads by SRCP

To determine with certainty that the SRCP does not contain double counted reads, we ran SRCP on each of the sides: R1 and R2. Next, we extracted the SRCP reported reads and filtered to look at the true circs only. We compare the list of read names which are associated to R1 as well as to R2. None of the reads appear in both lists.

### Differential expression analysis

We normalized the circular reads to the number of aligned reads in the sample. We used DESeq2 [47] to detect the differentially expressed circs. We combined the results from all pipelines into one data frame and filtered out circRNAs that had less than two reads. We then performed the analysis and selected the circRNAs with adjusted *p* value < 0.05 as significantly differently expressed.

### Ago2 HITS-CLIP re-analysis

We downloaded the data from GEO (GSE52082) and realigned it to human genome version Hg38 after trimming linker and adapter. SRCP was run using 5 base pair seeds and the brain circRNA list used as a reference. We retrieved miRNA abundance analysis from [49]. BigWig visualization files were generated with bamCoverage with a binSize of 5.

We expanded the miRNA binding site prediction in the detected circRNA backsplice junction reads using TargetScan7.0. For this, we extracted the reads supporting splice junctions from SAM files generated by SRCP. Then we run TragetScan 7.0 using *Homo Sapiens* miRNA family information.

### RNA extraction from *Drosophila* heads and RT-qPCR

Total RNA was prepared from female fly heads (day 1 and 21 post eclosion, D. melanogaster Canton-S) using TRI Reagent (Sigma, Aldrich) according to the manufacturer’s protocol. RNA was DNase treated (DNase I, NEB) and cDNA derived from this RNA (iScript Select cDNA synthesis Kit, Bio-Rad. Random Priming, following the manufacturer’s instructions) was utilized as a template for quantitative real-time PCR performed with the C1000 Thermal Cycler Bio-Rad. The PCR mixture contained Taq polymerase (SYBR green Bio-Rad). Cycling parameters were as follows: 95 °C for 3 min, followed by 40 cycles of 95 °C for 10 s, 55 °C for 10 s, and 72 °C for 30 s. Fluorescence intensities were plotted versus the number of cycles by using an algorithm provided by the manufacturer. All the primers used in this assay are listed in Additional file 6.

### RNaseR libraries

Human Adult Normal Brain Total RNA (R1234035-50), Human Adult Normal Cerebellum Total RNA (R1234039-50), Rat Brain Total RNA (R1434035-50), Monkey (Rh) Brain (R1534035-50) Total RNA, and Human Blood Total RNA (custom made) were obtained by BioChain.

One C57BL/6 male and one female were sacrificed at p21, different brain regions were harvested (cerebellum, cortex, olfactory bulb, brain stem, and thalamus) by the Nelson’s lab at Brandeis University. The RNA was extracted with TRI reagent (Sigma, Aldrich), followed by Zymo RNA cleaning and concentrator -25 Kit (Zymo), according to the manufacturers’ instruction. The RNA was treated with DNase (DNase I, NEB).

We collected and dissected 10-day-old *D. melanogaster* (Canton-S) heads and brains. Total RNA was extracted with TRI reagent (Sigma, Aldrich), followed by DNase (DNase I, NEB) treatment.

Ribodepletion was performed on 5 μg of total RNA with Ribo-Zero Gold rRNA Removal Kit according to the manufacturer’s instruction. Degradation of rRNA was checked by Tape Station. Ribodepleted RNA was treated with 3 units of RNaseR (Lucigen) per 1 μg of total RNA or with buffer only (Mock) for 15 min at 37 °C. The RNA was immediately extracted with TRI reagent (Sigma, Aldrich) according to the manufacturer’s instruction. For Human Blood Total RNA, only 2 μg of total RNA were rRNA-depleted and then treated with RNaseR as indicated above. For *D. melanogaster* samples, 2 μg of total RNA was rRNA-depleted with a homemade protocol and then treated with RNaseR as indicated above. Recovered RNA was used to prepare a total RNA library, adapting the protocol from [56]. Specifically, we prepared one library for the RNaseR-treated samples and one for the mock ones. Briefly, RNA was fragmented for 3 min at 92 °C in FAstAP 2X buffer (Thermo Fisher). Subsequently, samples were dephosphorylated with FastAP enzyme (Thermo Fisher), the first linker was ligated, and samples belonging to the same library were pulled together. Retro-transcription was performed using Affinity Script (Agilent). After second ligation, we performed PCR to add the external barcode. For Human Blood RNA samples and *D. melanogaster* samples, libraries were prepared using the NEXTFLEX® Rapid Directional RNA-Seq Kit 2.0 by PerkinElmer, following the manufacturer’s instructions. The samples were sequenced by Novogene (Novogene Corporation Inc. 8801 Folsom BLVD, Suite 290, Sacramento, CA 95826) with Hiseq-4000.

## Supplementary Information

**Additional file 1: Figures S1-S8.**

**Additional file 2: Supplementary tables. 1-5**.

**Additional file 3.** Correlations between Biological Replicas.

**Additional file 4.** List of validated circRNAs in the different species.

**Additional file 5.** AGO-HITS CLIP clusters and miRNA seed across backsplicing junctions of circRNAs found to be bound to AGO2.

**Additional file 6.** Oligonucleotides utilized in this study.

**Additional file 7.** Review history.

## Data Availability

SRCP is open-source under an MIT license and publicly available on Github (https://github.com/avigayel/SRCP) [57] and Zenodo (10.5281/zenodo.5497918) [58]. Total RNA and RNAse-R data from multiple species generated in this study were deposited in GEO under accession number GSE154616 [59]. Previously published total RNA and RNaseR from fly heads data used in this study are available at Gene Expression Omnibus under accession GSE55872 [44]. GSM1347830, GSM1347831, GSM1347838, GSM1347839, GSM1347842, GSM1347843, GSM1347834, and GSM1347835. Gene Expression Omnibus. https://www.ncbi.nlm.nih.gov/geo/query/acc.cgi?acc=GSE55872). Female fly head aging was retrieved from modENCODE project [32] SRR1197279, SRR1197275, SRR1197273, SRR1197274, SRR1197472, SRR1197473, SRR1197474, and SRR1197362. Gene Expression Omnibus. https://trace.ncbi.nlm.nih.gov/Traces/sra?study=SRP001696 2009). Previously published Human AGO2-HITS-CLIP data is available at Gene Expression Omnibus under accession GSE52082 [49] Gene Expression Omnibus. https://www.ncbi.nlm.nih.gov/geo/query/acc.cgi?acc=GSE52082 2014). Total RNA and RNAseR samples from human, macaque, and mouse brain were previously published and are available at BIG Data Center, Beijing Institute of Genomics (BIG), Chinese Academy of Science ID PRJCA000751 [48]. CRR026085, CRR026084 , CRR026043 , CRR026042 , CRR026004 , CRR026003. Beijing Institute of Genomics (BIG). https://ngdc.cncb.ac.cn/bioproject/browse/PRJCA000751 2019).

## References

[1] Hanan M, Soreq H, Kadener S (2017). CircRNAs in the brain. RNA Biol.

[2] Ebbesen KK, Hansen TB, Kjems J (2017). Insights into circular RNA biology. RNA Biol.

[3] Barrett SP, Salzman J (2016). Circular RNAs: analysis, expression and potential functions. Development.

[4] Patop IL, Wust S, Kadener S (2019). Past, present, and future of circRNAs. EMBO J.

[5] Petkovic S, Muller S (2015). RNA circularization strategies in vivo and in vitro. Nucleic Acids Res.

[6] Rybak-Wolf A, Stottmeister C, Glazar P, Jens M, Pino N, Giusti S, Hanan M, Behm M, Bartok O, Ashwal-Fluss R (2015). Circular RNAs in the mammalian brain are highly abundant, conserved, and dynamically expressed. Mol Cell.

[7] Veno MT, Hansen TB, Veno ST, Clausen BH, Grebing M, Finsen B, Holm IE, Kjems J (2015). Spatio-temporal regulation of circular RNA expression during porcine embryonic brain development. Genome Biol.

[8] You X, Vlatkovic I, Babic A, Will T, Epstein I, Tushev G, Akbalik G, Wang M, Glock C, Quedenau C, Wang X, Hou J, Liu H, Sun W, Sambandan S, Chen T, Schuman EM, Chen W (2015). Neural circular RNAs are derived from synaptic genes and regulated by development and plasticity. Nat Neurosci.

[9] Gruner H, Cortes-Lopez M, Cooper DA, Bauer M, Miura P (2016). CircRNA accumulation in the aging mouse brain. Sci Rep.

[10] Cortes-Lopez M, Gruner MR, Cooper DA, Gruner HN, Voda AI, van der Linden AM, Miura P (2018). Global accumulation of circRNAs during aging in Caenorhabditis elegans. BMC Genomics.

[11] Memczak S, Jens M, Elefsinioti A, Torti F, Krueger J, Rybak A, Maier L, Mackowiak SD, Gregersen LH, Munschauer M, Loewer A, Ziebold U, Landthaler M, Kocks C, le Noble F, Rajewsky N (2013). Circular RNAs are a large class of animal RNAs with regulatory potency. Nature.

[12] Hansen TB, Jensen TI, Clausen BH, Bramsen JB, Finsen B, Damgaard CK, Kjems J (2013). Natural RNA circles function as efficient microRNA sponges. Nature.

[13] Lustig Y, Barhod E, Ashwal-Fluss R, Gordin R, Shomron N, Baruch-Umansky K, Hemi R, Karasik A, Kanety H (2014). RNA-binding protein PTB and microRNA-221 coregulate AdipoR1 translation and adiponectin signaling. Diabetes.

[14] Holdt LM, Stahringer A, Sass K, Pichler G, Kulak NA, Wilfert W, Kohlmaier A, Herbst A, Northoff BH, Nicolaou A, Gäbel G, Beutner F, Scholz M, Thiery J, Musunuru K, Krohn K, Mann M, Teupser D (2016). Circular non-coding RNA ANRIL modulates ribosomal RNA maturation and atherosclerosis in humans. Nat Commun.

[15] Legnini I, Di Timoteo G, Rossi F, Morlando M, Briganti F, Sthandier O, Fatica A, Santini T, Andronache A, Wade M (2017). Circ-ZNF609 is a circular RNA that can be translated and functions in myogenesis. Mol Cell.

[16] Pamudurti NR, Bartok O, Jens M, Ashwal-Fluss R, Stottmeister C, Ruhe L, Hanan M, Wyler E, Perez-Hernandez D, Ramberger E, Shenzis S, Samson M, Dittmar G, Landthaler M, Chekulaeva M, Rajewsky N, Kadener S (2017). Translation of CircRNAs. Mol Cell.

[17] Yang Y, Fan X, Mao M, Song X, Wu P, Zhang Y, Jin Y, Yang Y, Chen LL, Wang Y, Wong CCL, Xiao X, Wang Z (2017). Extensive translation of circular RNAs driven by N(6)-methyladenosine. Cell Res.

[18] Piwecka M, Glazar P, Hernandez-Miranda LR, Memczak S, Wolf SA, Rybak-Wolf A, Filipchyk A, Klironomos F, Cerda Jara CA, Fenske P (2017). Loss of a mammalian circular RNA locus causes miRNA deregulation and affects brain function. Science.

[19] Reddy Pamudurti N, Konakondla-Jacob VV, Krishnamoorthy A, Ashwal-Fluss R, Bartok O, Wust S, et al. An in vivo knockdown strategy reveals multiple functions for circMbl. bioRxiv. 2018:483271.

[20] Pamudurti NR, Patop IL, Krishnamoorthy A, Ashwal-Fluss R, Bartok O, Kadener S (2020). An in vivo strategy for knockdown of circular RNAs. Cell Discov.

[21] Hanan M, Simchovitz A, Yayon N, Vaknine S, Cohen-Fultheim R, Karmon M, Madrer N, Rohrlich TM, Maman M, Bennett ER, Greenberg DS, Meshorer E, Levanon EY, Soreq H, Kadener S (2020). A Parkinson’s disease CircRNAs resource reveals a link between circSLC8A1 and oxidative stress. EMBO Mol Med.

[22] Matboli M, Shafei AE, Ali MA, Ashry AM, Kamal KM, Agag MA, Reda I, Tash EF (2018). Ali M: circRNAs (hsa_circ_00156, hsa_circ _000224, and hsa_circ _000520) are novel potential biomarkers in hepatocellular carcinoma. J Cell Biochem.

[23] Hansen TB, Veno MT, Damgaard CK, Kjems J (2016). Comparison of circular RNA prediction tools. Nucleic Acids Res.

[24] Jakobi T (2018). Dieterich C: deep computational circular RNA analytics from RNA-seq data. Methods Mol Biol.

[25] Szabo L, Salzman J (2016). Detecting circular RNAs: bioinformatic and experimental challenges. Nat Rev Genet.

[26] You X, Conrad TO (2016). Acfs: accurate circRNA identification and quantification from RNA-Seq data. Sci Rep.

[27] Cheng J, Metge F, Dieterich C (2016). Specific identification and quantification of circular RNAs from sequencing data. Bioinformatics.

[28] Otto C, Stadler PF, Hoffmann S (2014). Lacking alignments? The next-generation sequencing mapper segemehl revisited. Bioinformatics.

[29] Zhang XO, Wang HB, Zhang Y, Lu X, Chen LL, Yang L (2014). Complementary sequence-mediated exon circularization. Cell.

[30] Szabo L, Morey R, Palpant NJ, Wang PL, Afari N, Jiang C, Parast MM, Murry CE, Laurent LC, Salzman J (2015). Statistically based splicing detection reveals neural enrichment and tissue-specific induction of circular RNA during human fetal development. Genome Biol.

[31] Wang K, Singh D, Zeng Z, Coleman SJ, Huang Y, Savich GL, He X, Mieczkowski P, Grimm SA, Perou CM, MacLeod JN, Chiang DY, Prins JF, Liu J (2010). MapSplice: accurate mapping of RNA-seq reads for splice junction discovery. Nucleic Acids Res.

[32] Westholm JO, Miura P, Olson S, Shenker S, Joseph B, Sanfilippo P, Celniker SE, Graveley BR, Lai EC (2014). Genome-wide analysis of Drosophila circular RNAs reveals their structural and sequence properties and age-dependent neural accumulation. Cell Rep.

[33] Gao Y, Wang J, Zhao F (2015). CIRI: an efficient and unbiased algorithm for de novo circular RNA identification. Genome Biol.

[34] Zeng X, Lin W, Guo M, Zou Q (2017). A comprehensive overview and evaluation of circular RNA detection tools. PLoS Comput Biol.

[35] Hansen TB (2018). Improved circRNA identification by combining prediction algorithms. Front Cell Dev Biol.

[36] Meng X, Hu D, Zhang P, Chen Q, Chen M. CircFunBase: a database for functional circular RNAs. Database (Oxford). 2019;2019. 10.1093/database/baz003. 10.1093/database/baz003 PMC6360206 30715276

[37] Glazar P, Papavasileiou P, Rajewsky N (2014). circBase: a database for circular RNAs. RNA.

[38] Suzuki H, Zuo Y, Wang J, Zhang MQ, Malhotra A, Mayeda A (2006). Characterization of RNase R-digested cellular RNA source that consists of lariat and circular RNAs from pre-mRNA splicing. Nucleic Acids Res.

[39] Panda AC, De S, Grammatikakis I, Munk R, Yang X, Piao Y, Dudekula DB, Abdelmohsen K, Gorospe M (2017). High-purity circular RNA isolation method (RPAD) reveals vast collection of intronic circRNAs. Nucleic Acids Res.

[40] Xiao MS, Wilusz JE (2019). An improved method for circular RNA purification using RNase R that efficiently removes linear RNAs containing G-quadruplexes or structured 3’ ends. Nucleic Acids Res.

[41] Gao Y, Zhang J, Zhao F (2018). Circular RNA identification based on multiple seed matching. Brief Bioinform.

[42] Jeck WR, Sorrentino JA, Wang K, Slevin MK, Burd CE, Liu J, Marzluff WF, Sharpless NE (2013). Circular RNAs are abundant, conserved, and associated with ALU repeats. RNA.

[43] Salzman J, Gawad C, Wang PL, Lacayo N, Brown PO (2012). Circular RNAs are the predominant transcript isoform from hundreds of human genes in diverse cell types. PLoS One.

[44] Ashwal-Fluss R, Meyer M, Pamudurti NR, Ivanov A, Bartok O, Hanan M, Evantal N, Memczak S, Rajewsky N (2014). Kadener S: circRNA biogenesis competes with pre-mRNA splicing. Mol Cell.

[45] Gao Y, Zhang J, Zhao F (2017). Circular RNA identification based on multiple seed matching. Brief Bioinform.

[46] Li M, Xie X, Zhou J, Sheng M, Yin X, Ko EA, Zhou T, Gu W (2017). Quantifying circular RNA expression from RNA-seq data using model-based framework. Bioinformatics.

[47] Love MI, Huber W, Anders S (2014). Moderated estimation of fold change and dispersion for RNA-seq data with DESeq2. Genome Biol.

[48] Ji P, Wu W, Chen S, Zheng Y, Zhou L, Zhang J, Cheng H, Yan J, Zhang S, Yang P, Zhao F (2019). Expanded expression landscape and prioritization of circular RNAs in mammals. Cell Rep.

[49] Boudreau RL, Jiang P, Gilmore BL, Spengler RM, Tirabassi R, Nelson JA, Ross CA, Xing Y, Davidson BL (2014). Transcriptome-wide discovery of microRNA binding sites in human brain. Neuron.

[50] Agarwal V, Bell GW, Nam JW, Bartel DP. Predicting effective microRNA target sites in mammalian mRNAs. Elife. 2015;4. 10.7554/eLife.05005. 10.7554/eLife.05005 PMC4532895 26267216

[51] Xin R, Gao Y, Wang R, Kadash-Edmondson KE, Liu B, Wang Y, Lin L (2021). Xing Y: isoCirc catalogs full-length circular RNA isoforms in human transcriptomes. Nat Commun.

[52] Rahimi Karim VMT, Dupont DM, View ORCID Profile Kjems, Jørgen (2019). Nanopore sequencing of full-length circRNAs in human and mouse brains reveals circRNA-specific exon usage and intron retention.

[53] Zheng Y, Ji P, Chen S, Hou L, Zhao F (2019). Reconstruction of full-length circular RNAs enables isoform-level quantification. Genome Med.

[54] Quinlan AR, Hall IM (2010). BEDTools: a flexible suite of utilities for comparing genomic features. Bioinformatics.

[55] Langdon WB (2015). Performance of genetic programming optimised Bowtie2 on genome comparison and analytic testing (GCAT) benchmarks. BioData Min.

[56] Shishkin AA, Giannoukos G, Kucukural A, Ciulla D, Busby M, Surka C, Chen J, Bhattacharyya RP, Rudy RF, Patel MM, Novod N, Hung DT, Gnirke A, Garber M, Guttman M, Livny J (2015). Simultaneous generation of many RNA-seq libraries in a single reaction. Nat Methods.

[57] Rabin A, Ashwal-Fluss R, Patop IL, Jajoo A, Shenzis S, Carmel L, Kadener S (2021). SRCP: a comprehensive pipeline for accurate annotation and quantification of circRNAs.

[58] Rabin A, Zaffagni M, Ashwal-Fluss R, Patop IL, Jajoo A, Shenzis S, Carmel L, Kadener S (2021). SRCP: a comprehensive pipeline for accurate annotation and quantification of circRNAs.

[59] Rabin A, Zaffagni M, Ashwal-Fluss R, Patop IL, Jajoo A, Carmel L, Kadener S (2021). SRCP: a comprehensive pipeline for accurate annotation and quantification of circRNAs. GSE1544616. Gene Expression Omnibus.

