# Supplementary material for: SRCP: a comprehensive pipeline for accurate annotation and quantification of circRNAs
Source: Genome Biol. 2021 Sep 23;22:277. doi: 10.1186/s13059-021-02497-7 (PMC8459468; doi:10.1186/s13059-021-02497-7)
Supplement: Supplementary file 1 — Additional file 1: Figures S1-S8. [file 13059_2021_2497_MOESM1_ESM.pdf]

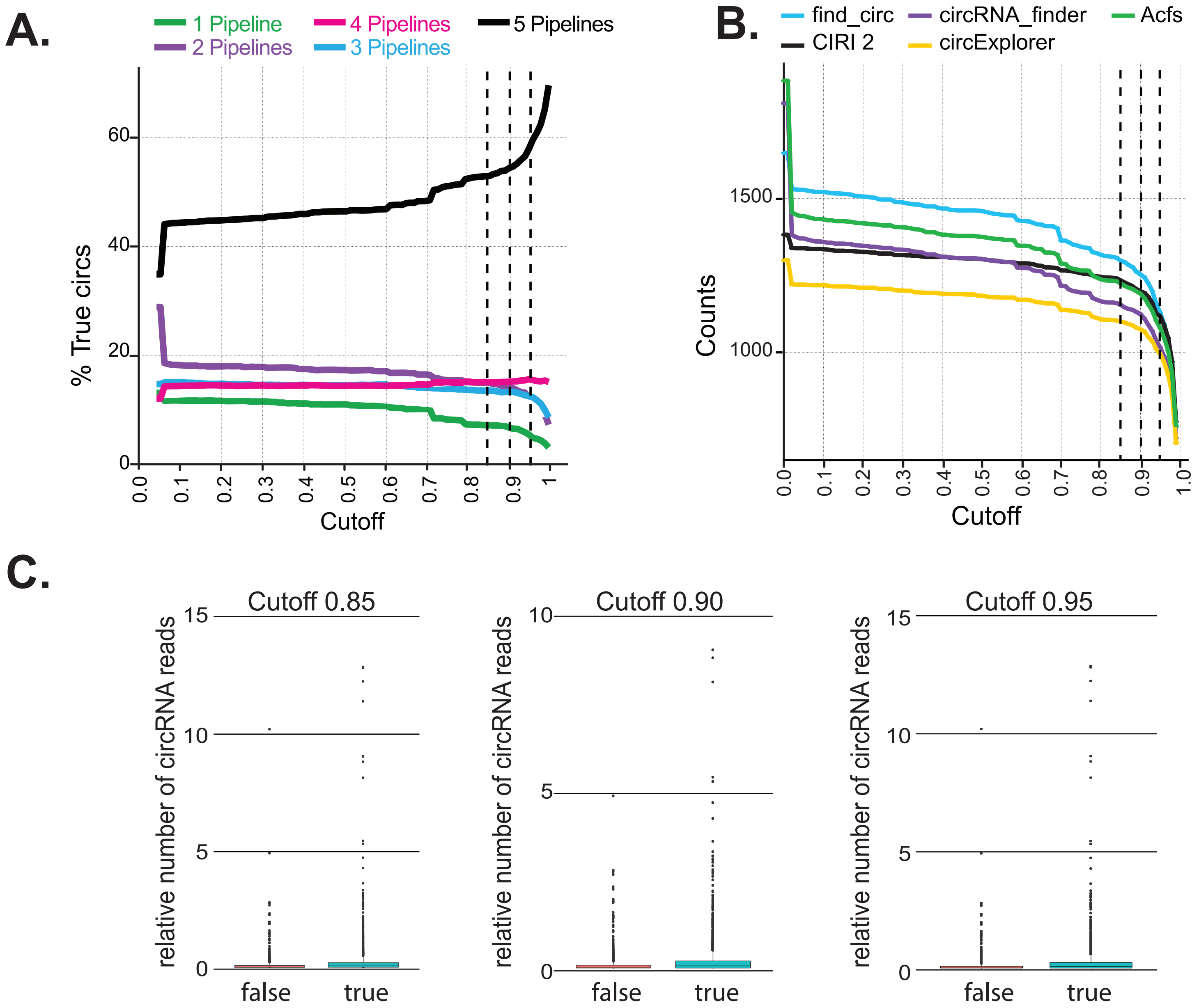

D.

# pipelines discovered the circs	1	2	3	4	5
# circs	16	4	3	2	4

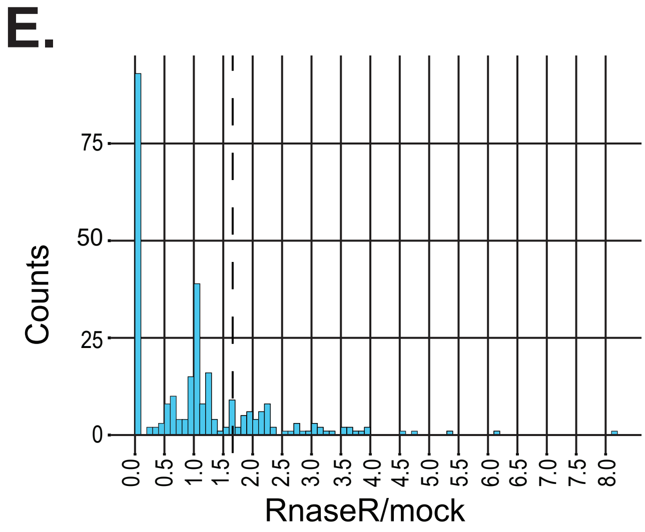

Figure S1

Figure S1: Removing the false positives circRNAs

A. The percent of circRNAs identified as "true" positives as a function of the cut-off for circRNAs identified by 1, 2, 3, 4, or 5 of the pipelines used. The dotted lines indicate three potential threshold/cut off (0.85, 0.9, or 0.95). The cutoff is defined as the fraction of linear mRNAs that would be included in the true list. **B.** Number of true-positive circRNAs identified by each pipeline using different cut-off values. **C.** Boxplots showing the distribution of expression of the true and false circRNA that are identified in the indicated cutoff values. **D.** Table indicating whether the most highly expressed 29 circRNAs in the false list were detected by 1, 2, 3, or 4 pipelines. **E**. RNaseR/mock ratio of the pipeline-specific circRNAs (those identified only by one pipeline). The dashed line indicates the cutoff value. All circles in the right of the line are annotated as "true" and the circles in the left as "false".

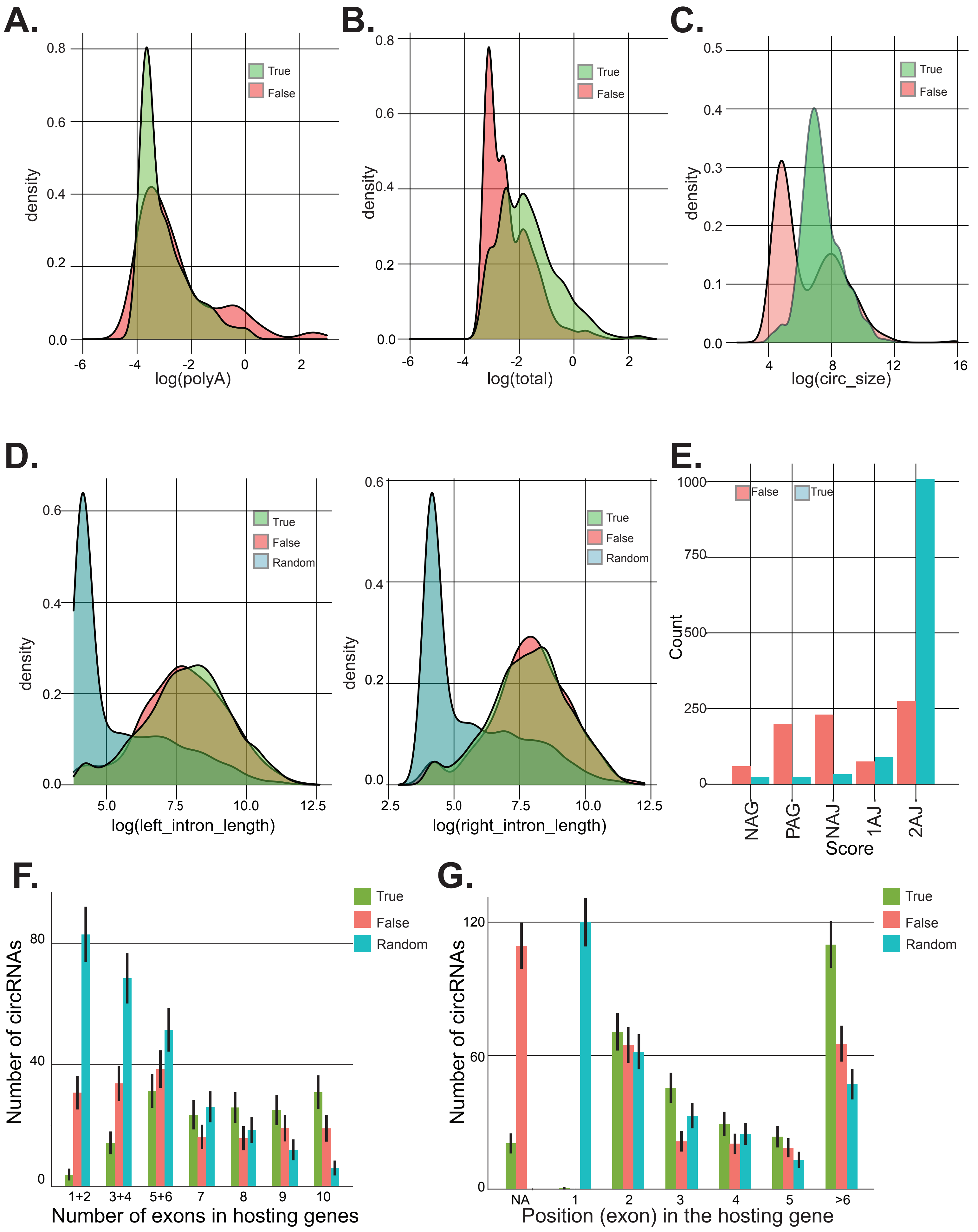
A.
density
log(polyA)
True
False
B.
density
log(total)
True
False
C.
density
log(circ_size)
True
False
D.
density
log(left_intron_length)
log(right_intron_length)
True
False
Random
E.
Count
Score
False
True
NAG
PAG
NAJ
1AJ
2AJ
F.
Number of circRNAs
Number of exons in hosting genes
True
False
Random
1+2
3+4
5+6
G.
Number of circRNAs
Position (exon) in the hosting gene
True
False
Random
NA
>6

Figure S2

Figure S2: Certain genomic features distinguish circRNAs.

A. PolyA distribution of the true-positive and false-positive circRNAs in a *D. melanogaster* library. **B.** Expression distribution of true-positive and false-positive circRNAs. **C.** Distribution of the circRNA size in true-positive and false-positive circRNAs. **D.** Distributions of the intron lengths flanking true-positive and false-positive circRNAs and randomly selected exons in the upstream direction (left) and downstream direction (right). **E.** Number of indicated annotations for exons of true-positive and false-positive circRNAs and randomly selected exons. NAG -not annotated gene; PAG -partially annotated gene; NAJ - no annotated junction; 1AJ - one annotated junction; 2AJ -two annotated junctions. **F.** Number of exons in the hosting gene for true-positive and false-positive circRNAs and randomly selected exons. To calculate the number of exons in a circRNA hosting gene, we selected a group of 300 True circRNAs, 300 False circRNAs and 300 random circles. We calculated the number of exons in each group and repeated this process 1,000 times. The error bars represent the variability of the number of circs that were detected with X number of exons across the iterations. **G.** Position of the circularizable exon/s in the hosting gene for True-positive circRNAs, False-positive circRNAs, and randomly selected exons. Iteration analysis and error bars were performed as for F.

A

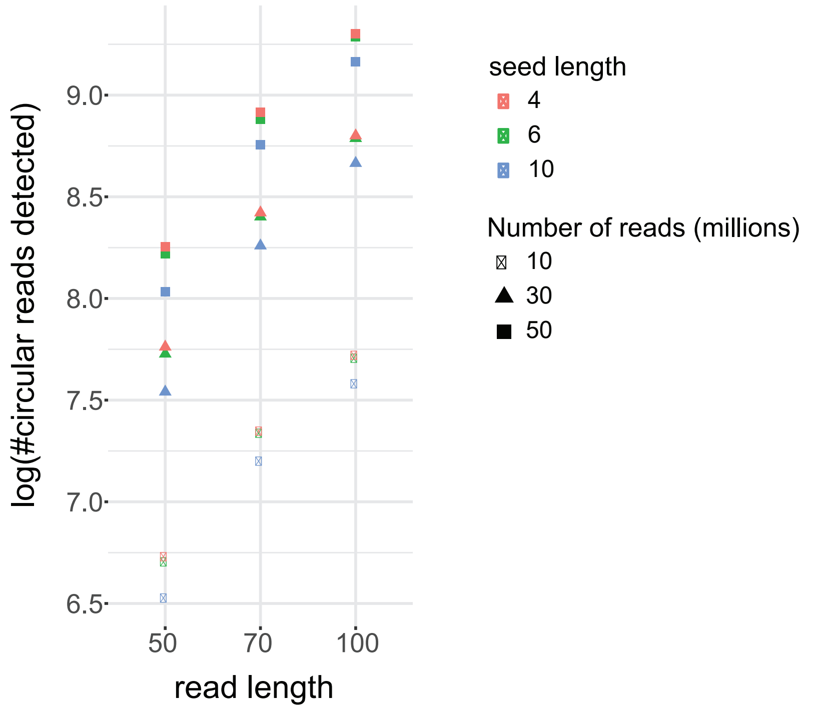

log(#circular reads detected)
9.0
8.5
8.0
7.5
7.0
6.5
50
70
100
read length
seed length
4
6
10
Number of reads (millions)
10
30
50

B

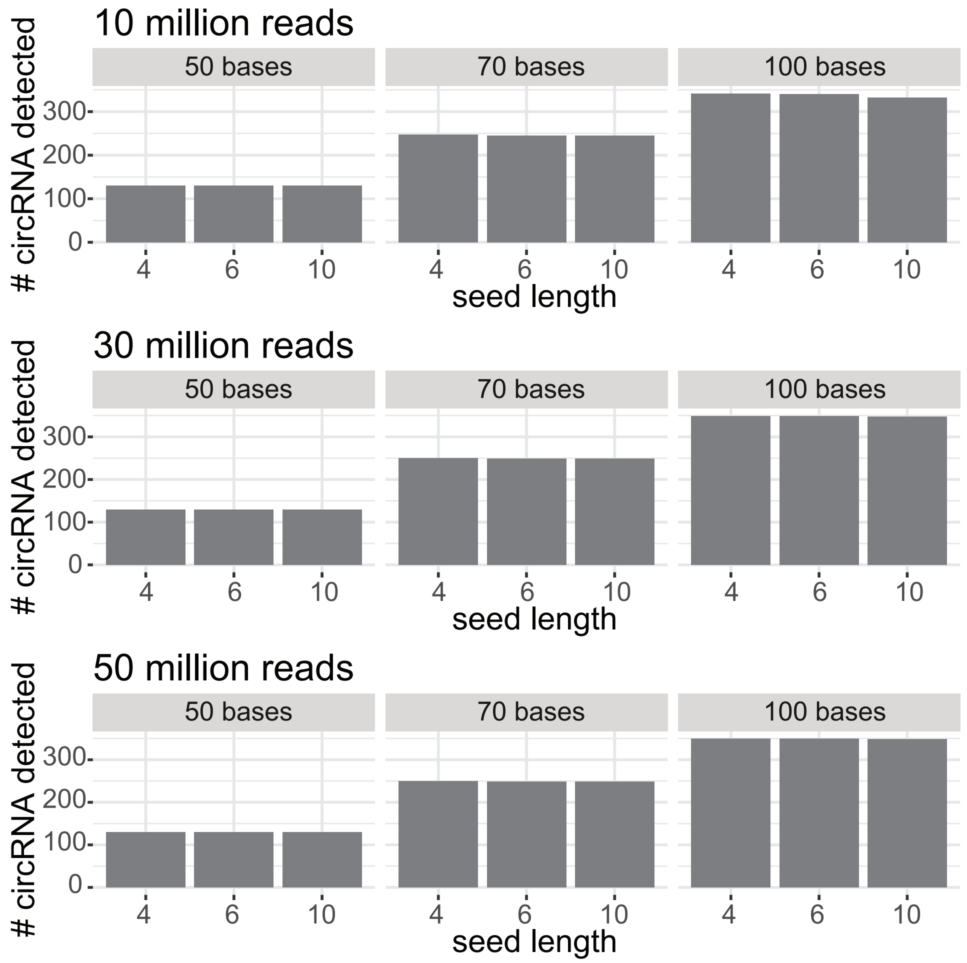

10 million reads
30 million reads
50 million reads
50 bases
70 bases
100 bases
circRNA detected
300
200
100
0
4
6
10
seed length

Figure S3

Figure S3: SRCP detects circular reads in samples with low coverage depth.

A. Number of circular reads detected by SRCP for the different read lengths and sample depths utilizing different seed matching. **B.** Number of circRNAs detected in the different samples using different lengths of seeds.

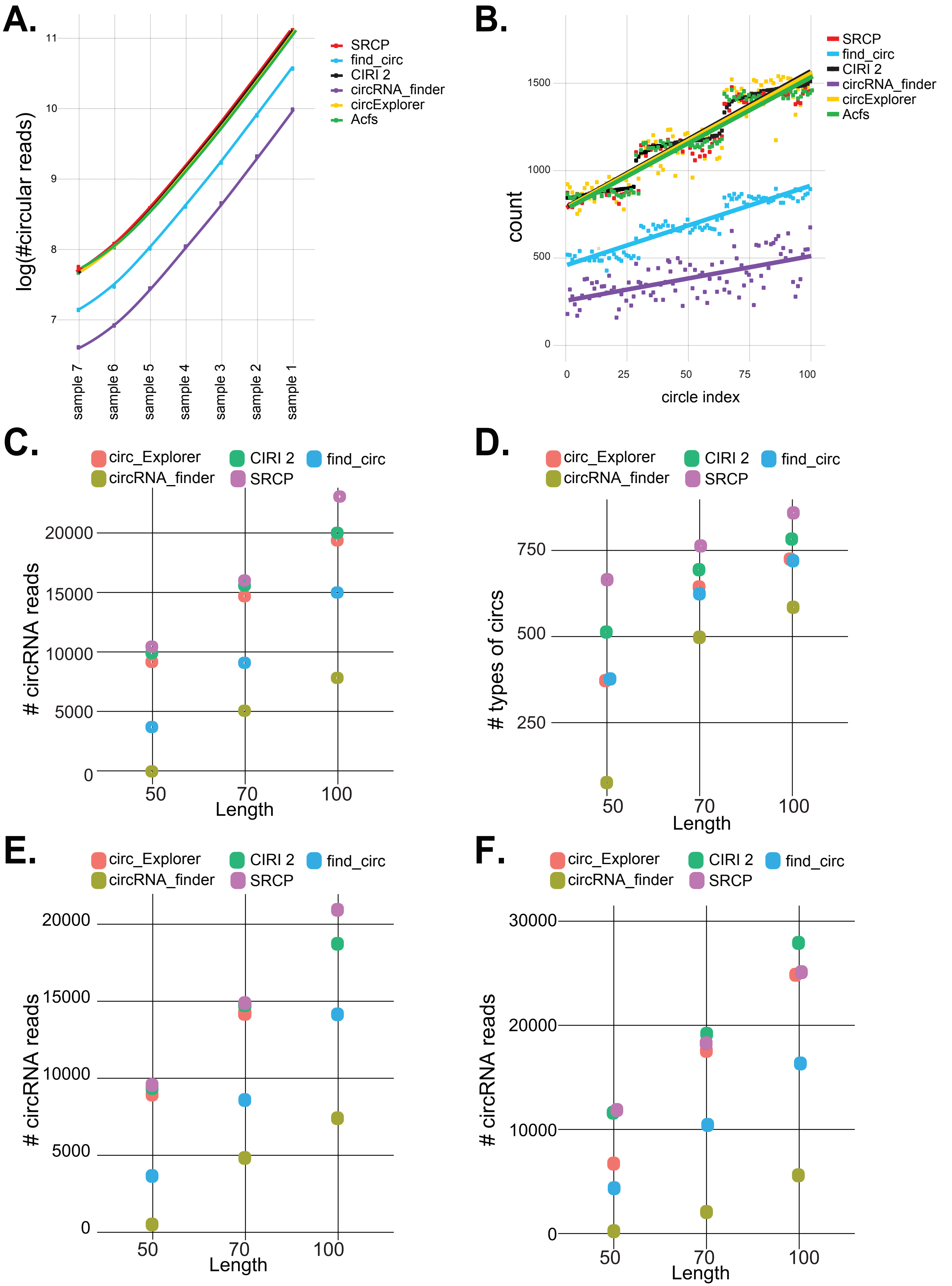
A.
log(#circular reads)
11
10
9
8
7
sample 7
sample 6
sample 5
sample 4
sample 3
sample 2
sample 1
SRCP
find_circ
CIRI 2
circRNA_finder
circExplorer
Acfs
B.
count
1500
1000
500
0
0
25
50
75
100
circle index
C.
circ_Explorer
CIRI 2
find_circ
circRNA_finder
SRCP
circRNA reads
20000
15000
10000
5000
0
50
70
100
Length
D.
types of circs
750
500
250
E.
F.
30000
20000
10000

Figure S4

Figure S4: circRNAs can be accurately quantified using seed matching in simulated and real data.

A. Number of circRNA reads found by SRCP and the indicated circRNA-identification pipelines in each of the simulated samples. B**.** Number of circRNA reads found by each pipeline for the 100 most highly expressed circRNAs in the simulation. **C.** Total number of circRNA RNAseq reads for true circRNAs detected by the different pipelines in one of the samples (SRR1197473) in the intact PE reads (100 bases long) or after computationally truncate them to 50 or 70 bases long. **D.** Number of types of True circRNAs identified by the different pipelines in the SRR1197473 sample in the whole (100 bases) or truncated (to 50 or 70 bases) reads. **E.** Similar to C and D but we plotted the number of reads originated from the True-common circRNAs. **F.** Total number of True circRNA RNAseq reads for the different pipelines when analyzing the two reads (R1 and R2) of the SRR1197473 sample independently (as single end reads), As in D-F, we have done the analysis in the whole read (100 bases) or after it was truncated to be 50 or 70 bases long.

A.

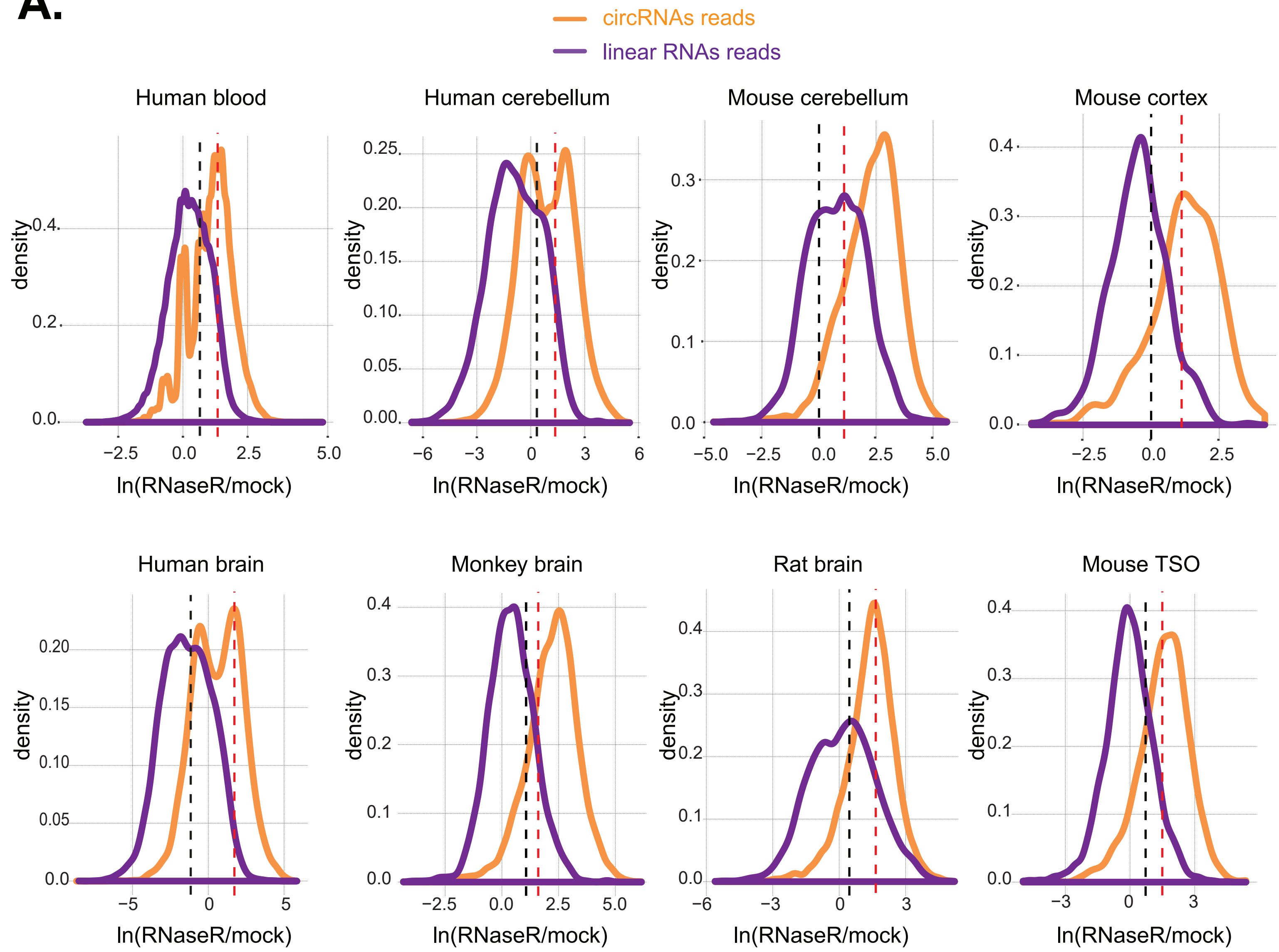

circRNAs reads
linear RNAs reads
Human blood
Human cerebellum
Mouse cerebellum
Mouse cortex
Human brain
Monkey brain
Rat brain
Mouse TSO
density
ln(RNaseR/mock)

Figure S5

Figure S5: Validation of True/*bona fide* circRNAs in four mammalian species.

RNaseR/mock ratio distribution in the indicated specie and tissue. The data in orange represent the circular junctions and that in violet the linear junctions. Black dotted line indicates utilized cutoff. Red dotted line indicates cutoff using the 5-fold enrichment criteria.

A.

B.

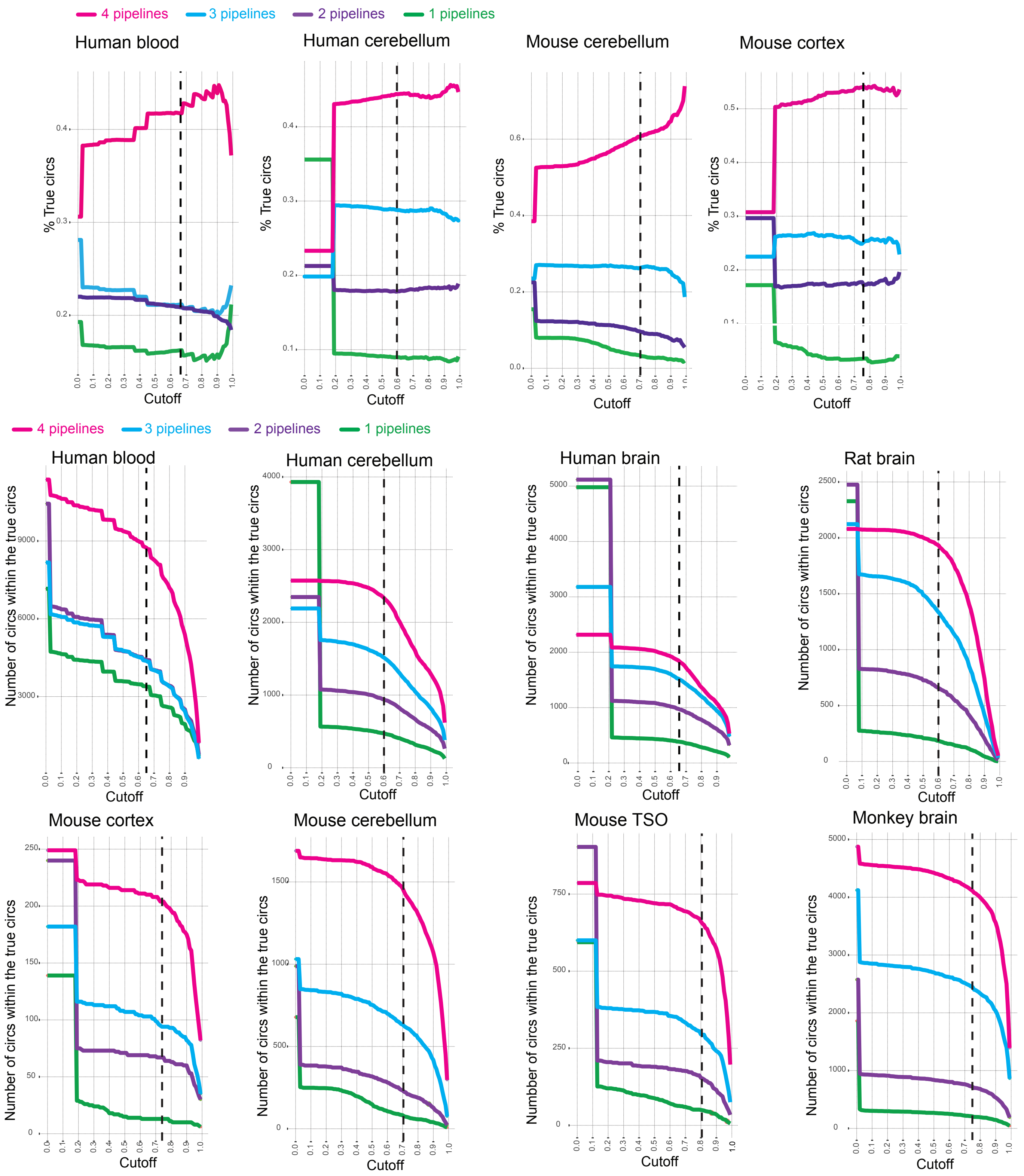

C.

		1 pipeline	2 pipelines	3 pipelines	4 pipelines
Human Cerebellum	True	543	1277	2111	3355
	False	2672	802	252	31
Human Blood	True	860	3428	7775	10392
	False	3143	6403	4678	3759
Mouse Cortex	True	16	93	158	403
	False	51	71	16	2
Mouse Cerebellum	True	87	245	654	1488
	False	589	740	381	202

Figure S6

Figure S6: circRNAs detected by multiple pipelines are generally more resistant to RNaseR. **A.** The percentage of circRNAs identified as "true" positives as a function of the cut-off for circRNAs identified by 1, 2, 3, or 4 of the pipelines used in the indicated species and tissue. **B.** The number of circRNAs identified as "true" positives as a function of the cut-off for circRNAs identified by 1, 2, 3, or 4 of the pipelines used in the indicated species and tissue. **C.** Table summarizing the number of pipelines that identify the sets of true and false circRNAs identified from the indicated tissues and species. For building this table we utilized the thresholds marked in Figure S6A and S6B as a dotted line and indicated in the table in 5C.

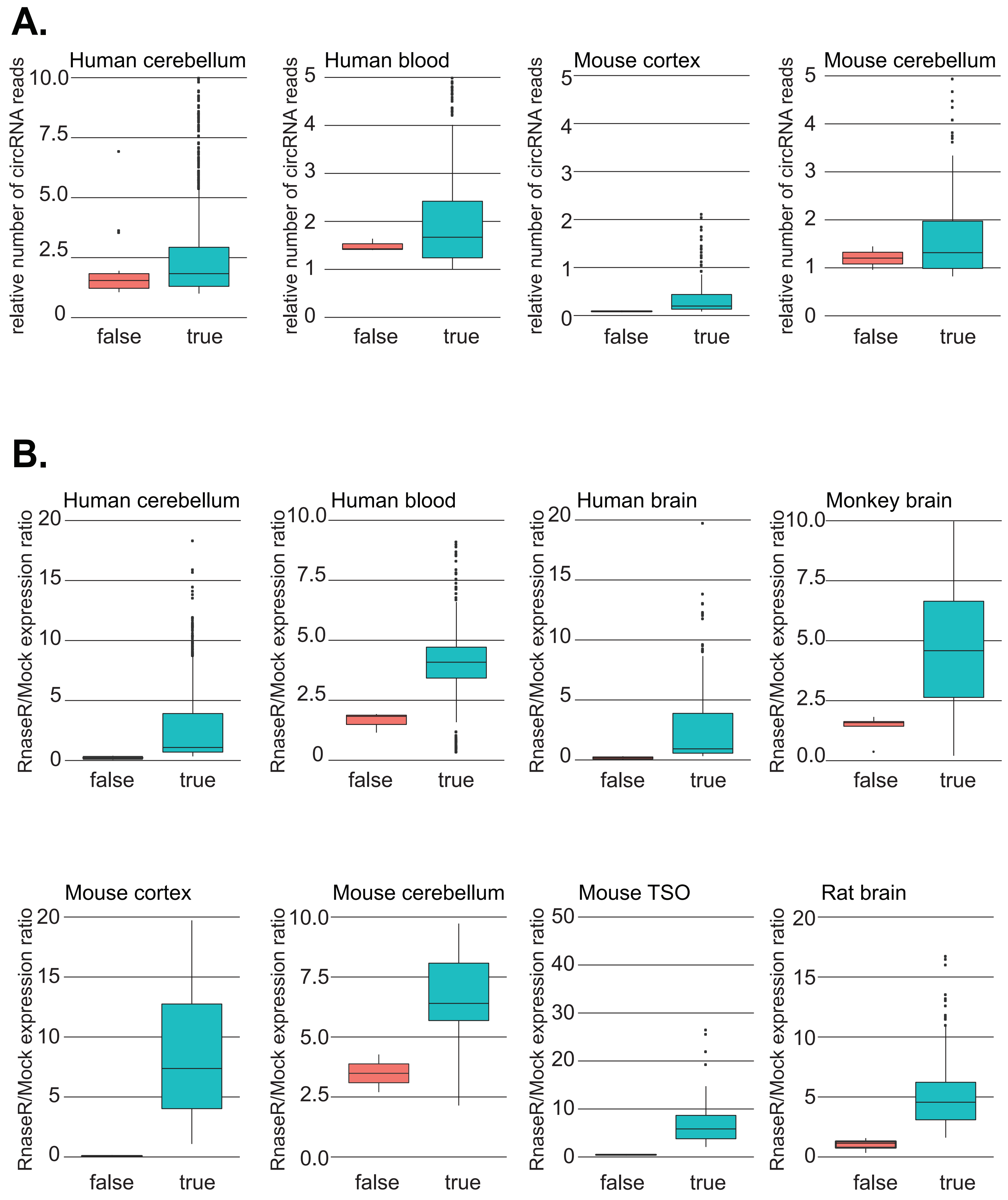
A.
Human cerebellum
Human blood
Mouse cortex
Mouse cerebellum
relative number of circRNA reads
false
true
B.
Human cerebellum
Human blood
Human brain
Monkey brain
Mouse cortex
Mouse cerebellum
Mouse TSO
Rat brain
RnaseR/Mock expression ratio
false
true

Figure S7

Figure S7: circRNAs in the false group tend to be very lowly expressed.

A. Boxplots showing the distribution of expression of the true and false circRNA that are identified in the indicated tissues after the corrections mentioned in the text. **B.** Boxplots showing the distribution of RNaseR/mock expression ratio for the true and false circRNAs defined as in A.

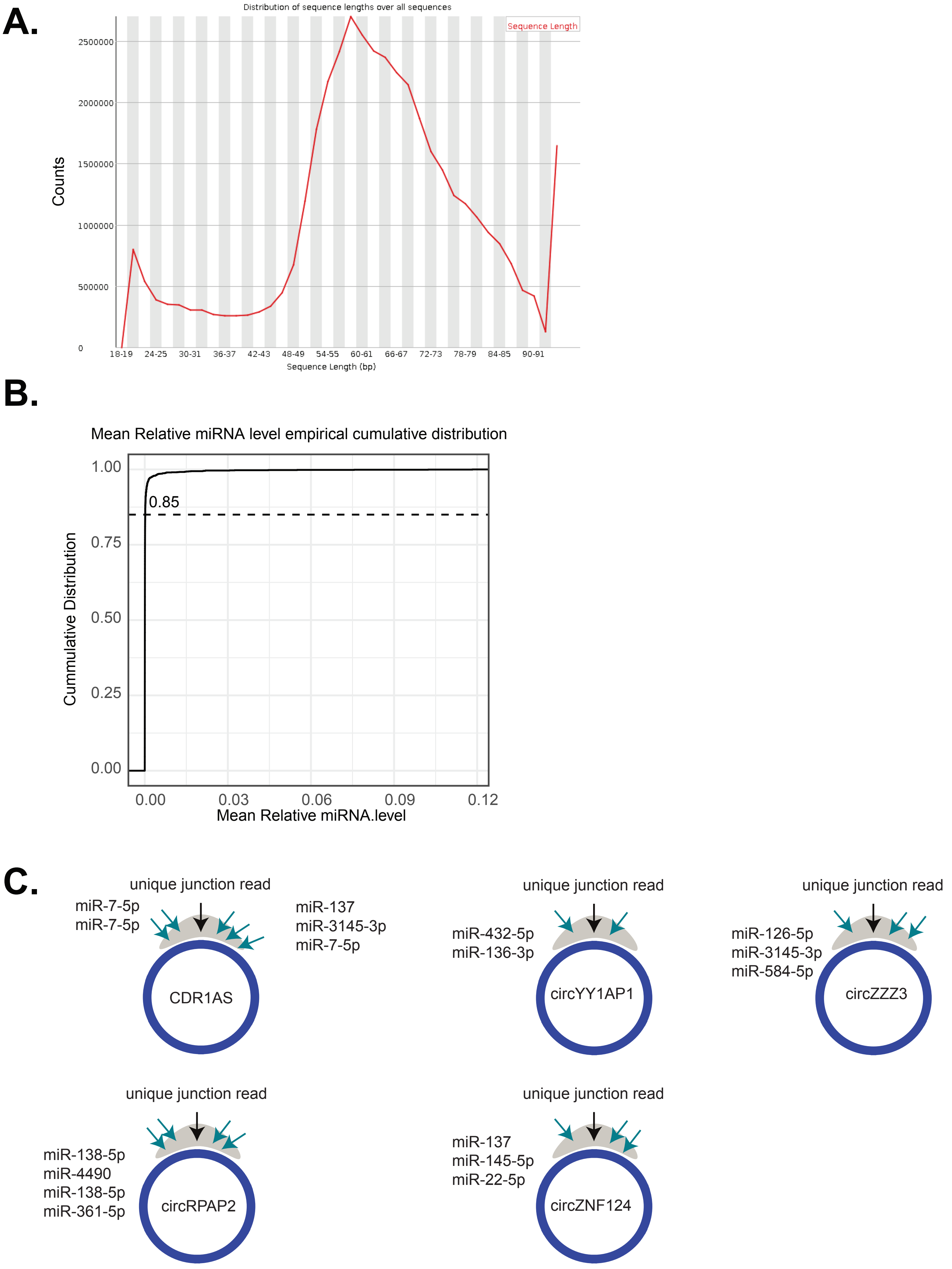

A.
Distribution of sequence lengths over all sequences
Sequence Length
Counts
2500000
2000000
1500000
1000000
500000
0
18-19
24-25
30-31
36-37
42-43
48-49
54-55
60-61
66-67
72-73
78-79
84-85
90-91
Sequence Length (bp)
B.
Mean Relative miRNA level empirical cumulative distribution
Cummulative Distribution
1.00
0.75
0.50
0.25
0.00
0.85
0.00
0.03
0.06
0.09
0.12
Mean Relative miRNA.level
C.
unique junction read
miR-7-5p
miR-7-5p
miR-137
miR-3145-3p
miR-7-5p
CDR1AS
unique junction read
miR-432-5p
miR-136-3p
circYY1AP1
unique junction read
miR-126-5p
miR-3145-3p
miR-584-5p
circZZZ3
unique junction read
miR-138-5p
miR-4490
miR-138-5p
miR-361-5p
circRPAP2
unique junction read
miR-137
miR-145-5p
miR-22-5p
circZNF124

Figure S8

Figure S8: circRNAs bind to AGO2 in the human brain.

A. Example of read length distribution after linker and adapter removal from the utilized dataset. **B.** Cumulative distribution plot of miRNA abundance. In line the cut-off of top 15%. **C.** miRNA binding sites in reads spanning the back-splice junction for CDR1AS, circYIAP1, circZZZ3, circRPA2, and circZNF124.
