# Supplementary material for: SRCP: a comprehensive pipeline for accurate annotation and quantification of circRNAs
Source: Genome Biol. 2021 Sep 23;22:277. doi: 10.1186/s13059-021-02497-7 (PMC8459468; doi:10.1186/s13059-021-02497-7)
Supplement: Supplementary file 2 — Additional file 2: Supplementary tables. 1-5. [file 13059_2021_2497_MOESM2_ESM.docx]

## Tables

### Table S1: Cut-off and performance

| Number of pipelines that identified the putative circRNA | Cut-off | | | | | |
| --- | --- | --- | --- | --- | --- | --- |
|  | 0.9 | | 0.95 | | 0.99 | |
|  | TRUE | FALSE | TRUE | FALSE | TRUE | FALSE |
| 1 | 4.19% | 40.29% | 2.31 % | 38.60% | 0.36% | 33.77% |
| 2 | 12.44% | 15.82% | 9.66 % | 16.93% | 5.03% | 18.47% |
| 3 | 11.74% | 27.52% | 10.62% | 25.92% | 6.5% | 22.70% |
| 4 | 13.84% | 8.99% | 14.13% | 9.9% | 13.7% | 12.37% |
| 5 | 54.76% | 7.35% | 63.25% | 8.63% | 74.3% | 12.66% |

### Table S2: Simulated samples and number of reads

| Sample | batch1 | batch2 | batch3 | batch4 | batch5 | batch6 – control |
| --- | --- | --- | --- | --- | --- | --- |
| 1 | 1000 | 800 | 600 | 400 | 200 | 50 |
| 2 | 500 | 400 | 300 | 200 | 100 | 50 |
| 3 | 250 | 200 | 150 | 100 | 50 | 50 |
| 4 | 125 | 100 | 75 | 50 | 25 | 50 |
| 5 | 63 | 50 | 37 | 25 | 13 | 50 |
| 6 | 32 | 25 | 19 | 13 | 7 | 50 |
| 7 | 16 | 13 | 10 | 7 | 4 | 50 |

### Table S3: Differentially expressed circRNAs identified using SRCP and different circRNA-identification pipelines

| Pipeline | Total | True | False |
| --- | --- | --- | --- |
| SRCP | 102 | 102 | 0 |
| Find_circ | 87 | 77 | 10 |
| CIRI | 71 | 60 | 11 |
| circRNA_finder | 60 | 55 | 4 |
| circExplorer | 80 | 73 | 7 |
| Acfs | 78 | 74 | 4 |
| Saifish-cir | 21 | 21 | 0 |

### Table S4: Number of reads in atlas samples

| **Species** | **Region** | **Treatment** | **#Reads** | **GEO accession number** |
| --- | --- | --- | --- | --- |
| Human | brain | Mock | 102,765,873 | GSM4675884  GSM4996434 |
| Human | brain | RNaseR | 26,436,966 | GSM4675885 |
| Human | Cerebellum | Mock | 92,883,214 | GSM4675882  GSM4996433 |
| Human | Cerebellum | RNaseR | 29,147,311 | GSM4675883 |
| Human | Blood | Mock | 219,866,200 | GSM4675896  GSM4675897 |
| Human | Blood | RNaseR | 285,469,788 | GSM4996440  GSM4996441 |
| Mouse | Cortex | Mock | 33,359,590 | GSM4675892  GSM4996438 |
| Mouse | Cortex | RNaseR | 34,810,751 | GSM4675893 |
| Mouse | Cerebellum | Mock | 59,450,971 | GSM4675890  GSM4996437 |
| Mouse | Cerebellum | RNaseR | 196,711,754 | GSM4675891 |
| Mouse | olfactory bulb, brain stem, and thalamus | Mock | 37,445,301 | GSM4675894  GSM4996439 |
| Mouse | olfactory bulb, brain stem, and thalamus | RNaseR | 76,127,642 | GSM4675895 |
| Monkey | brain | Mock | 91,229,854 | GSM4675886  GSM4996435 |
| Monkey | brain | RNaseR | 151,069,976 | GSM4675887 |
| Rat | brain | Mock | 137,413,300 | GSM4675888  GSM4996436 |
| Rat | brain | RNaseR | 73,845,076 | GSM4675889 |

**Table S5. Number of circRNAs identified by the indicated number of pipelines and found as true utilizing the SRCP approach but found less than 5-fold enriched in the RNaseR samples.**

| **Tissue/# of pipelines** | **1** | **2** | **3** | **4** | **TOTAL** |
| --- | --- | --- | --- | --- | --- |
| **Mouse Cerebellum** | 14 | 24 | 38 | 70 | 146 |
| **Mouse Cortex** | 8 | 57 | 111 | 304 | 480 |
| **Mouse TSO** | 32 | 88 | 171 | 362 | 653 |
| **Human Brain** | 383 | 978 | 1942 | 3087 | 6390 |
| **Human Blood** | 586 | 2269 | 4488 | 6699 | 14042 |
| **Human Cerebellum** | 357 | 890 | 1529 | 2398 | 5174 |
| **Monkey Brain** | 152 | 316 | 636 | 777 | 1881 |
| **Rat Brain** | 93 | 317 | 653 | 775 | 1838 |
